# AI-Enhanced Social Robotic Versus Computer-Based Virtual Patients for Clinical Reasoning Training in Medical Education: Observational Crossover Cohort Study

**DOI:** 10.2196/82541

**Published:** 2025-11-27

**Authors:** Alexander Borg, Jonathan Schiött, William Ivegren, Cidem Gentline, Viking Huss, Anna Hugelius, Benjamin Jobs, Fabricio Espinosa, Mini Ruiz, Samuel Edelbring, Carina Georg, Gabriel Skantze, Ioannis Parodis

**Affiliations:** 1 Division of Rheumatology, Department of Medicine Solna, Karolinska Institutet, Karolinska University Hospital, and Center for Molecular Medicine (CMM) Stockholm Sweden; 2 Division of Clinical Epidemiology, Department of Medicine Solna, Karolinska Institutet and Karolinska University Hospital Stockholm Sweden; 3 Department of Clinical Science, Intervention and Technology, Karolinska Institutet Stockholm Sweden; 4 School of Education, Culture and Communication, Mälardalen University Västerås Sweden; 5 School of Health Sciences, Örebro University Örebro Sweden; 6 Department of Neurobiology, Care Sciences and Society, Karolinska Institutet Stockholm Sweden; 7 Division of Speech Music and Hearing, Royal Institute of Technology (KTH) Stockholm Sweden; 8 Department of Rheumatology, Faculty of Medicine and Health, Örebro University Örebro Sweden

**Keywords:** virtual patients, medical education, clinical reasoning, large language models, social robotics, artificial intelligence, educational technology

## Abstract

**Background:**

Virtual patient (VP) simulations can be used to practice clinical reasoning (CR) in controlled learning environments. Traditional computer-based VP platforms often lack the authenticity and interactivity required for effective CR training. Artificial intelligence (AI)–enhanced social robotic VPs can enhance realism and engagement; however, quantitative evidence comparing them with conventional VP platforms remains limited.

**Objective:**

We compared medical students’ experience of an AI-enhanced social robotic versus a conventional computer-based VP platform regarding the extent to which the design characteristics of the respective platform facilitate CR skill training.

**Methods:**

This observational crossover cohort study involved 178 sixth-semester medical students at Karolinska Institutet, Stockholm, Sweden (response rate: 42.3%; 178 of 421 invited students; Spring 2024-Spring 2025), who experienced both a large language model–enhanced social robotic VP platform supporting dialogue (social artificial intelligence–enhanced robotic interface [SARI]) and a conventional computer-based VP platform (virtual interactive case [VIC]) during their clinical rotation within rheumatology. Platform order was determined by clinical rotation scheduling. VP design was evaluated using a validated questionnaire across 5 domains: authenticity, professional approach, coaching quality, learning effects, and overall judgment. Students’ CR training preferences were assessed using categorical responses and a Visual Analogue Scale, where a lower score favored SARI and a score of 5 indicated equal preference between platforms.

**Results:**

SARI outperformed VIC across all 5 VP design domains. Students rated SARI higher for authenticity (median 4.0, IQR 3.5-4.5 vs 3.0, IQR 2.5-3.5; *P*<.001), professional approach (median 4.5, IQR 4.0-4.8 vs 4.0, IQR 3.5-4.5; *P*<.001), coaching quality (median 4.3, IQR 4.0-4.7 vs 4.0, IQR 3.7-4.7; *P*<.001), learning effect (median 4.4, IQR 4.0-5.0 vs 4.0, IQR 3.5-4.5; *P*<.001), and overall judgment (median 5.0, 4.0-5.0 vs 4.0, IQR 4.0-5.0; *P*<.001). Students strongly preferred SARI for CR training (72% vs 14%; odds ratio [OR] 27.1, 95% CI 14.3-53.7; *P*<.001), with Visual Analogue Scale scores confirming this preference (median 3.0, IQR 2.0-5.0; *P*<.001). Preferences were consistent across most subgroups (sex, prior VP experience, and platform order); in 2 subgroups, the difference was not significant, that is, students with prior VP experience (62% vs 38%; OR 2.6; 95% CI 0.8-8.9; *P*=.11) and students first introduced to VIC (55% vs 45%; OR 1.5; 95% CI 0.7-2.9; *P*=.33).

**Conclusions:**

Our findings provide the first quantitative evidence that AI-enhanced social robotic VPs offer superior design characteristics than conventional computer-based platforms for CR training in medical education. These results support the use of AI-driven social robots for VP simulations to better prepare medical students for real clinical encounters, and warrant future research on objective CR skill outcomes and long-term transfer to clinical practice. Unlike previous qualitative studies examining each platform separately, this study provides the first quantitative comparison of design characteristics between AI-enhanced social robotic and conventional computer-based VPs.

## Introduction

Virtual patients (VP) are digital educational modalities that enable learners within health professions to interact with patient cases for learning purposes [1]. These educational tools complement real-life patient interactions by allowing health care learners to practice gathering medical histories and making clinical and diagnostic decisions in controlled, safe environments [2,3]. VPs can be developed using various technologies and are often used for clinical reasoning (CR) training purposes [4,5]. It has recently been demonstrated that VP-based educational tools can effectively improve medical students’ CR skills across multiple domains, including problem-solving and data gathering [6]. However, successful implementation depends on instructional design and quality features that support active learning [7]. While VPs offer standardized training opportunities, medical students report that conventional computer-based implementations often lack authenticity, potentially limiting their educational impact [8,9].

CR represents a fundamental cognitive process that guides diagnostic and management decisions in clinical practice [10-12]. While CR is important for patient safety and clinical outcomes [13] and has been recommended to be explicitly addressed in health professions education [14], traditional training approaches may not adequately capture the dynamic and complex nature of clinical decision-making [15]. VPs offer unique opportunities to train and assess CR skills using standardized, repeatable scenarios that simulate real clinical encounters [16,17]. However, many current VP platforms focus primarily on easily assessable CR components, with limited research examining how VP design characteristics can support the development of more complex aspects such as clinical presentations, generation of hypotheses, and justification of diagnostic decisions [18].

Recent advances in artificial intelligence (AI), particularly the introduction of large language models (LLMs), have enhanced VP platforms and their ability to provide sophisticated clinical scenarios and realistic patient responses that better simulate the complexity of real CR challenges [19-22]. LLMs demonstrate potential to transform medical education through interactive simulations, individualized tutoring, and personalized feedback [23,24]. When LLMs are integrated into physical embodied agents such as social robotic interfaces, they have the potential to deliver multimodal interactions that enhance the cognitive engagement required for effective CR skill development [21]. Emerging evidence suggests that physical embodied AI enables multimodal dynamic learning and real-time feedback through direct environmental interaction, potentially offering advantages over screen-based learning [25].

Our previous qualitative research demonstrated that sixth-semester medical students perceived AI-enhanced social robotic interfaces as more clinically authentic than traditional computer-based VPs for CR training [22]. While this qualitative evidence suggests that AI-enhanced social robotic VPs provide more authentic learning experiences compared with traditional computer-based platforms, no quantitative studies have hitherto examined whether these perceived advantages translate into measurably better VP design characteristics that support CR skill development.

Given the critical importance of CR skills in clinical practice and the challenges of providing authenticity using traditional VPs, quantitative evaluation is essential to determine the effectiveness of emerging VP technologies [26,27]. This study aimed to compare medical students’ experience of an AI-enhanced social robotic versus a conventional computer-based VP platform, regarding the extent to which the design characteristics of the respective platform facilitate training of CR skills.

## Methods

### Overview

We conducted an observational crossover cohort study to compare VP design elements that support CR skill training in medical education in an LLM-empowered social robotic platform, that is, an in-house developed social artificial intelligence–enhanced robotic interface (SARI) [20-22], and a computer-based VP platform, that is, the virtual interactive case system (VIC) [28]. This study reports according to the STROBE (Strengthening the Reporting of Observational Studies in Epidemiology) [29] guidelines for observational studies and the GREET (Guideline for Reporting Evidence-Based Practice Educational Interventions and Teaching) guidelines [30] for educational references.

All students experienced both VP platforms as a part of their clinical rotations within rheumatology. Platform order was determined by clinical rotation scheduling and practical logistics rather than random assignment. Each student served as their own control to minimize between-participant variability. We collected quantitative data using a questionnaire previously developed for VP platform evaluation (measuring authenticity, professional approach, coaching quality, learning effects, and overall judgment) within the context of CR [31], combined with additional evaluation items developed by our research team to assess the preference of each VP platform for CR training. The complete questionnaire is provided in Figure S1 in Multimedia Appendix 1.

### Study Design and Setting

The study was conducted at Karolinska Institutet (KI), a major academic medical university in Stockholm, Sweden. Data collection occurred during clinical rotations at the Division of Rheumatology, Karolinska University Hospital, between the spring term of 2024 and the spring term of 2025. The clinical rotations within rheumatology are mandatory for all medical students at KI and take place during the sixth semester of medical studies.

### Study Participants

Study participants comprised sixth-semester medical students enrolled in clinical courses at KI in Stockholm, Sweden. All medical students at KI participate in clinical rotations within rheumatology at the Karolinska University Hospital as a part of their curriculum. During these clinical rotations, students participate in an educational activity called “the virtual outpatient clinic,” where students encounter VPs.

We used convenience sampling, recruiting all sixth-semester medical students who completed their clinical rotation within rheumatology during the study period and consented to participate in the study. All students (N=421) were invited to participate by completing a survey after their educational experience with both VP platforms. A total of 178 agreed to participate, yielding a response rate of 42.3%. Students who did not complete the virtual outpatient clinic activity during the study period (eg, due to illness or absence for other reasons) were excluded from the study. Language proficiency was not an additional inclusion or exclusion criterion. However, students with insufficient English proficiency would have been unable to participate; no such cases were encountered. Participation was voluntary, and students provided written informed consent prior to enrolment, with the opportunity to withdraw at any time. Students received no reimbursement for participation.

### VP Case Development and Practice

The VP cases had been developed and implemented for the virtual outpatient clinic before the study commenced, following specific recommendations for VP case development. Those included (1) structured case design models with difficulty levels adapted to students’ curriculum, (2) incorporation of CR strategies such as differential diagnostic formulation, and (3) interactive elements in history-taking and physical examination options [32,33]. All cases were developed in English to also ensure accessibility for international exchange students. A total of 10 cases were developed: 5 cases were presented using SARI and 5 cases using VIC. One case was identical between the 2 platforms, which served the comparison between the 2 VP platforms during the early stages of their development [20,21]. Students interacted with 5 cases on one platform and 4 on the other, experiencing a total of 9 unique VPs. The unequal distribution of VP cases (5 cases on one platform and 4 cases on the other) was therefore to avoid repetition of the identical case rather than deliberate research design. This resulted in some student groups encountering 5 cases on SARI, and some encountering 5 cases on VIC, with this selection being solely based on scheduling. The cases included representative patient scenarios of rheumatological conditions supporting the students’ learning curriculum, including polymyalgia rheumatica, rheumatoid arthritis, psoriatic arthritis, ankylosing spondylitis, systemic lupus erythematosus, and Sjögren disease. A detailed description of the case development process and the case contents has been described elsewhere [22].

Each student attended the virtual outpatient clinic over a period of one and a half days, during which they encountered and interacted with all VP cases. Before training with the cases, the students received written information on generic medical history questions typically used at the rheumatology outpatient clinic to gather a structured history from patients and support diagnostic procedures (Figure S2 in Multimedia Appendix 1). The students were instructed to perform the VP sessions in pairs or small groups of 3 students to allow for interaction and active collaboration, which has been shown to favor CR skill training compared with experiencing the VP sessions alone [34]. Cases were initiated with a brief case presentation, which included the patient’s primary concern, age, and name. Next, the students explored the case environment in their preferred order. Cases were concluded when students perceived that they had gathered sufficient information to perform preliminary diagnostics and propose a suitable management plan for the VP. Students were allocated approximately 30 minutes per VP case interaction.

Following completion of each case, the students participated in case-specific follow-up seminars to discuss the case with a seminar leader, that is, a consultant rheumatologist at the Karolinska University Hospital, and pose questions. During these seminars, students were asked to summarize the VP case briefly in a structured manner to practice clinical communication and to propose their recommendations for further management. These seminar series comprised 2 to 3 student groups who had just performed the same VP case (ie, 4 to 9 students), and lasted approximately 15-20 minutes per case. While all students performed all 9 VP cases, platform order was determined by clinical rotation scheduling logistics. Of the 178 participants, 101 (56%) students began with SARI and 77 (44%) began with VIC on the first day of the virtual outpatient clinic. This distribution was not the result of random assignment but rather reflected practical scheduling for the clinical rotations. The students completed the virtual outpatient clinic assignment after finishing all VP cases along with their corresponding follow-up seminars.

### Social AI-Enhanced Robotic Interface

The embodiment of SARI consisted of a social robot from Furhat Robotics, which projects an animated face onto a semi-transparent face mask. The face mask is placed on a plastic head, which is connected to a mechanical neck that allows natural head movements and adjustments of gaze direction to facilitate interaction with multiple users simultaneously, using sensors [35]. Furthermore, the robot displays facial expressions and provides affective responses along with sophisticated gaze behavior to indicate emotions during interaction [36]. SARI combines the Furhat software development kit (FurhatSDK) with the OpenAI GPT-3.5 turbo LLM [37]. To limit the risk of unwanted AI hallucinations from SARI, we used prompts to generate dialogue responses using specific instructions, including a detailed patient description along with the 10 latest dialogue turns. SARI was constrained to provide clinically appropriate information consistent with the VP case description. The LLM was also prompted to generate facial expressions to match the emotional state of the VP during dialogue, selecting from available expressions in the FurhatSDK at specific anchor points during the dialogues [38]. A VP prompt example is provided in Figure S3 in Multimedia Appendix 1.

Before starting a VP case in SARI, students received brief written contextual information along with results from relevant laboratory tests with their corresponding reference values. SARI imposed no numerical limits on the questions students could ask during case interactions. Students could engage in natural language dialogue for as long as they deemed necessary to gather sufficient clinical information. A schematic illustration of the VP interaction between students and SARI is illustrated in Figure S4 in Multimedia Appendix 1.

### Virtual Interactive Case System

VIC is a web-accessible, computer-based VP platform where users freely interact with a VP case, while the initial presentation of the case and the conclusion remain fixed [39]. We incorporated interactive case elements primarily regarding medical history, physical examination options, laboratory tests, and relevant diagnostic imaging. There was no limit to the number of times students could revisit the prespecified questions or examination options. However, the students were encouraged to be selective in investigating information they deemed relevant to the case as more information became available. The cases concluded with students providing preliminary diagnoses and proposing management plans by selecting from multiple-choice responses.

### Ethical Considerations

The Swedish Ethical Review Authority approved this study prior to initiation (registration number: 2022-04437-01). The study was conducted in accordance with the Declaration of Helsinki and Swedish regulations. All participants provided written informed consent before enrollment. Students were explicitly informed that participation was voluntary and would not affect their academic standing or evaluation in any way. Participants had the right to withdraw their participation in the study at any time without providing reasons and without consequences for their education. All study data were collected pseudonymously through coded questionnaires. No personally identifiable information was recorded in the dataset. Questionnaire responses were only linked to pseudonymized participant codes. Data were stored securely on password-protected servers at KI, with access restricted to researchers directly involved in the study. All data handling procedures complied with the European General Data Protection Regulation. Students received no financial compensation or academic credits for their participation in the study. Participation was entirely voluntary beyond the mandatory educational virtual outpatient activity, which all students completed regardless of their participation in the study.

### Data Collection

Immediately after completion of the virtual outpatient clinic, students who had agreed to participate in this study completed a questionnaire to evaluate the VP platform design, with particular emphasis on CR training. The questionnaire consisted of the complete instrument for VP design evaluation developed and validated by Huwendiek et al [31], which uses Likert-scale data based on statements regarding the VP experience and has demonstrated good content validity and internal consistency. To this, we added 2 project-specific questions designed by our research group to capture preferences regarding VP platforms for CR training. These additional items had not undergone formal validation, as they were tailored to the objectives of this study. The adapted questionnaire underwent pilot testing within our team and with a small group of students to ensure clarity and comprehensibility before use in the study. Internal consistency of the adapted questionnaire was not assessed in our sample. The complete questionnaire is provided in Figure S1 in Multimedia Appendix 1.

Likert-scale data were divided into the following quantitative themes from the questionnaire by Huwendiek et al [31]: (1) authenticity of the patient encounter and the consultation, (2) professional approach in the consultation, (3) coaching during consultation, (4) learning effect of consultation, and (5) overall judgment of the case work-up. Within each theme, students evaluated a statement relating to their VP experience, for example, “while working through this case, I felt I had to make the same decisions a clinician would make in real life,” which was scored from 1 (strongly disagree) to 5 (strongly agree).

The two questions relating to the preferred VP platform were (1) “Overall, which of the platforms is preferable to you in relation to acquirement of clinical reasoning skills?” and (2) “On a scale from 0 to 10, where 0 is total preference for the social robot and 10 is total preference for the computer-based platform, how would you grade your preference of the virtual patient platforms compared with each other for acquirement of clinical reasoning skills?” The first question was answered using one of 3 responses: SARI, VIC, or equally preferred, while the second question had the structure of a Visual Analogue Scale (VAS), ranging between 0 and 10, where a lower score denoted a stronger preference for SARI, a higher score denoted a stronger preference for VIC, and a score of 5 denoted equal preference.

In addition to questions relating to the VP design and evaluations of the preferred platform for CR training, students provided information regarding their age and sex, whether they had any previous experience with VPs, and which platform they were first introduced to during the virtual outpatient clinic.

### Statistical Analysis

The Wilcoxon signed-rank test was used to compare responses to Likert-scale data from quantitative themes in the questionnaire described by Huwendiek et al [31] and VAS responses. For each theme, individual item scores were averaged to create a composite theme score for each student. Theme scores were next compared between platforms. VAS responses from the 2 platforms were compared with a hypothetical score of 5, denoting equal preference. The Fisher exact test with Monte Carlo simulation (10.000 iterations) was used to compare frequencies of categorical responses for VP platform preference based on students’ CR training experience. Results from Wilcoxon signed-rank tests are presented as medians and the corresponding IQR, test statistics (*W*), effect size (*r*), and *P* value. Results from Fisher exact tests are presented as frequencies and the corresponding percentage, odds ratio (OR), 95% CI, and *P* value.

Missing data were minimal across all variables. Demographic variables had no missing values except platform order (1 missing value, 0.56%). For Likert scale items evaluating VP design, missing data were 1.68% overall (range: 0%-3.91% per item; maximum 7 missing responses out of 178). VAS preference scores had 1.12% missing values (2 of 178 responses), and categorical platform preference had 1.68% missing values (3 of 178 responses).

Little’s MCAR test on Likert data indicated that the data may not be completely at random (*χ*^2^_108_=157.12; *P*=.001); however, given the very low proportion of missing data (<2% overall) and the paired nature of our crossover design, complete case analysis with pairwise deletion was used. No systematic differences in missingness across demographic groups were observed for any variable (all *P*>.05). The number of participants with available data is reported for each analysis (Tables 1 and 2). “Not applicable” responses (coded as 6 in the questionnaire; up to 9.5% for some items) were treated as valid response categories in frequency distributions but were excluded from statistical tests, as they do not represent a position on the agree-disagree scale. Our sample size was determined by the total number of sixth-semester medical students completing their clinical rotation in rheumatology during the study period. Post hoc power analysis indicated that this sample size provided >80% power to detect moderate effect sizes (Cohen *d*≥0.5) at of 0.05 for paired comparisons. All statistical analyses were performed using R (version 4.3.3; R Foundation for Statistical Computing, Vienna, Austria). Differences yielding *P* values <.05 were considered statistically significant.

**Table 1:** Comparison of median scores using the Wilcoxon signed-rank test within each question of the clinical reasoning questionnaire based on Likert-scale data. Comparison of median Likert scale scores (1=strongly disagree to 5=strongly agree) between social artificial intelligence–enhanced robotic interface (SARI) and virtual interactive case (VIC) for individual questionnaire items evaluating virtual patient design in an observational crossover cohort study of 178 sixth-semester medical students at Karolinska Institutet, Stockholm, Sweden, between the Spring of 2024 and the Spring of 2025. Students completed questionnaires evaluating both platforms after experiencing them during their clinical rotation within rheumatology. Five domains were assessed as follows: (1) authenticity of patient encounter, (2) professional approach in consultation, (3) coaching during consultation, (4) learning effect of consultation, and (5) overall judgment of case work-up. Data were analyzed using the Wilcoxon signed-rank test for paired comparisons. Missing data rates were low for all items (overall: 1.68%; range: 0%-3.91% across items). “Not applicable” responses were excluded from statistical analyses but included in frequency distributions (Tables S1-S4 in Multimedia Appendix 2). Data are presented as the median score (IQR), test statistic (W), and effect size (r). The total number of study participants was 178. In case of missing values, the number of participants with available data is indicated.

Theme and variable		SARI, median (IQR)	VIC, median (IQR)	*W*	*r*	*P* value
**Authenticity of patient encounter**						
	Q1^a^ (n=172)	4.0 (3.0-4.0)	3.0 (3.0-4.0)	5306	0.34	<.001^b^
	Q2 (n=173)	4.0 (3.0-4.0)	3.0 (2.0-3.0)	6936	0.58	<.001^b^
**Professional approach in the consultation**						
	Q1 (n=172)	5.0 (4.0-5.0)	4.0 (3.0-5.0)	4011	0.43	<.001^b^
	Q2 (n=173)	4.0 (4.0-5.0)	4.0 (4.0-5.0)	1378	0.28	<.001^b^
	Q3 (n=169)	4.0 (3.0-5.0)	4.0 (3.0-4.0)	2146	0.43	<.001^b^
	Q4 (n=171)	5.0 (4.0-5.0)	4.0 (4.0-5.0)	868	0.27	<.001^b^
**Coaching during consultation**						
	Q1 (n=174)	5.0 (4.0-5.0)	5.0 (4.0-5.0)	248	0.11	.119
	Q2 (n=158)	4.0 (4.0-5.0)	4.0 (3.3-5.0)	1123	0.29	<.001^b^
	Q3 (n=152)	4.0 (4.0-5.0)	4.0 (3.0-5.0)	1268	0.31	<.001^b^
**Learning effect of consultation**						
	Q1 (n=175)	4.0 (4.0-5.0)	4.0 (3.0-4.0)	1491	0.35	<.001^b^
	Q2 (n=173)	4.0 (4.0-5.0)	4.0 (3.0-4.0)	1515	0.39	<.001^b^
**Overall judgment of case work-up**						
	Q1 (n=175)	5.0 (4.0-5.0)	4.0 (4.0-5.0)	2351	0.35	<.001^b^

^a^Q: question.

^b^Statistically significant values.

**Table 2:** Comparison of median composite theme scores between platforms using the Wilcoxon signed-rank test. Composite scores were calculated by averaging each student’s responses within themes. Comparison of median composite theme between social artificial intelligence–enhanced robotic interface (SARI) and virtual interactive case (VIC) using the Wilcoxon signed-rank test in an observational crossover cohort study of 178 sixth-semester medical students at Karolinska Institutet, Stockholm, Sweden, between the Spring of 2024 and the Spring of 2025. Students completed questionnaires evaluating both platforms after experiencing them during their clinical rotation within rheumatology. Composite scores were calculated by averaging each student’s responses within the five themes of a validated virtual patient design characteristics questionnaire: (1) authenticity of patient encounter, (2) professional approach in consultation, (3) coaching during consultation, (4) learning effect of consultation, and (5) overall judgment of case work-up. Data were analyzed using the Wilcoxon signed-rank test for paired comparisons. Missing data rates were low for all items (overall: 1.68%; range: 0%-3.91% across items). “Not applicable” responses were excluded from statistical analyses but included in frequency distributions (Tables S1-S4 in Multimedia Appendix 2). Data are presented as the median score (IQR), test statistic (W), and effect size (r). The total number of study participants was 178. In case of missing values, the number of participants with available data is indicated.

Theme	SARI, median (IQR)	VIC, median (IQR)	*W*	*r*	*P* value
Authenticity of patient encounter (n=173)	4.0 (3.5-4.5)	3.0 (2.5-3.5)	9253	0.54	<.001^a^
Professional approach in the consultation (n=173)	4.5 (4.0-4.8)	4.0 (3.5-4.5)	6717	0.54	<.001^a^
Coaching during consultation (n=174)	4.3 (4.0-4.7)	4.0 (3.7-4.7)	4092.5	0.32	<.001^a^
Learning effect of consultation (n=175)	4.4 (4.0-5.0)	4.0 (3.5-4.5)	2589	0.42	<.001^a^
Overall judgment of case work-up (n=175)	5.0 (4.0-5.0)	4.0 (4.0-5.0)	2351	0.35	<.001^a^

^a^Statistically significant values.

## Results

### Demographic and Study-Specific Characteristics

Of the 178 students who participated in the questionnaire, 93 (52%) were women and 86 (48%) were men. Most students had no previous experience with VP platforms (150 students, 84%), while 29 (16%) reported prior experience. The mean age was 25.3 (SD 5.4) years. Regarding platform order, 101 (56%) students started with SARI, and 77 (43%) started with VIC.

### VP Platform Design Evaluation for Clinical Reasoning Training

**Figure 1:**
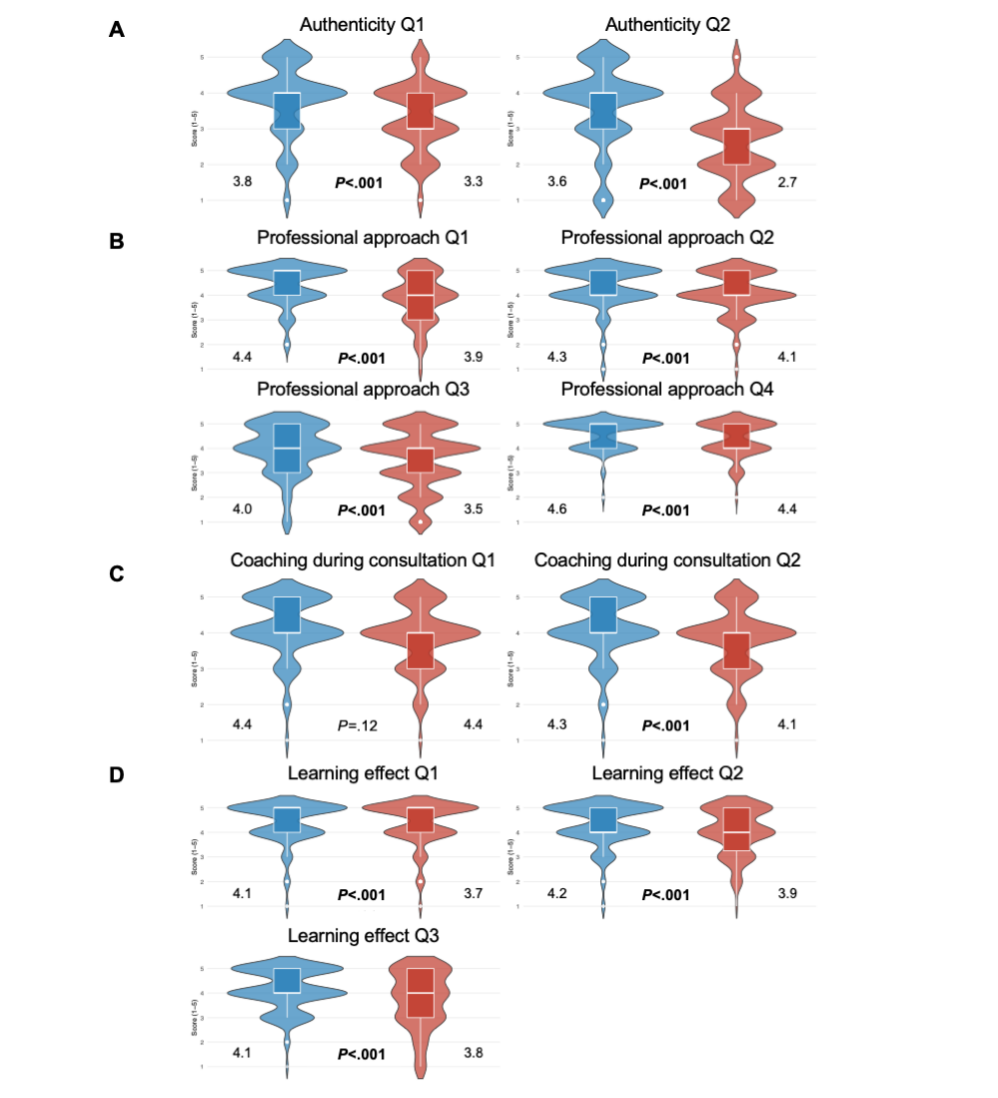
Violin plots illustrating results from the Wilcoxon signed-rank test on Likert-scale data (1=strongly disagree to 5=strongly agree) from student evaluations of virtual patient platform design with an emphasis on clinical reasoning training in an observational crossover cohort study of 178 sixth-semester medical students at Karolinska Institutet, Stockholm, Sweden, between the Spring of 2024 and the Spring of 2025. Students completed questionnaires evaluating both platforms after experiencing them during their clinical rotation within rheumatology. Comparisons between social artificial intelligence–enhanced robotic interface (blue color) and virtual interactive case (red color) are illustrated. Violin plots show score distributions; white boxes indicate the median and IQR, and numbers indicate mean scores. Panel A denotes theme A: authenticity of patient encounter (question 1: “I felt I had to make the same decisions a doctor would make in real life”; question 2: “I felt I was the doctor caring for this patient”). Panel B denotes theme B: professional approach in consultation (question 1: “I was actively engaged in gathering the information I needed”; question 2: “I was actively engaged in revising my reasoning as new information became available”; question 3: “I was actively engaged in creating a summary of the patient’s problems using medical terms”; question 4: “I was actively engaged in thinking about which findings supported or refuted each diagnosis I was considering”). Panel C denotes theme C: coaching during consultation (question 1: “I felt that the case was at the appropriate level of difficulty for my training”; question 2: “the questions I was asked while working through this case helped me to learn”; question 3: “the feedback I received was helpful in enhancing my diagnostic reasoning process”). Panel D denotes theme D: learning effect of consultation (question 1: “I feel better prepared to confirm a diagnosis and exclude differential diagnoses in a real patient with this complaint”; question 2: “I feel better prepared to care for a real patient with this condition”). Q: question.

**Figure 2:**
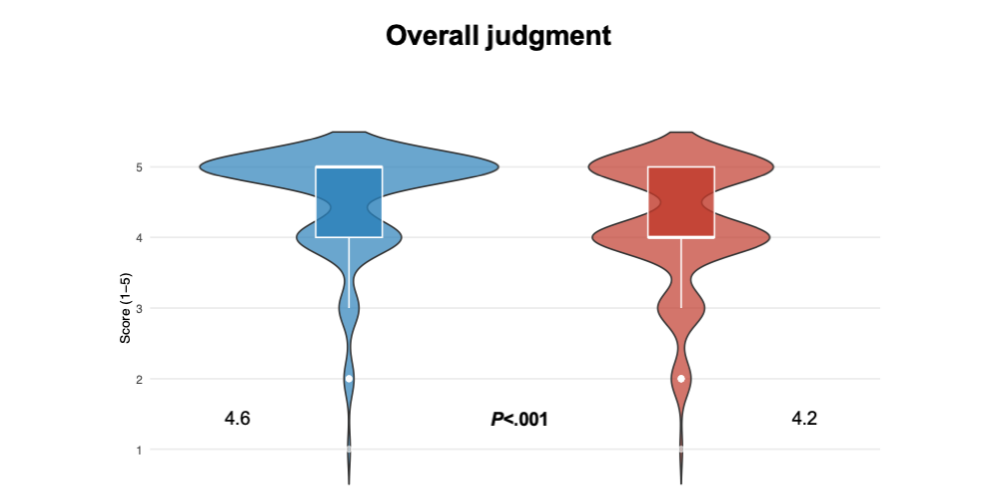
Violin plots illustrating results from the Wilcoxon signed-rank test on Likert-scale data (1=strongly disagree to 5=strongly agree) from student evaluations of virtual patient platform design with an emphasis on clinical reasoning training regarding the theme overall judgment in an observational crossover cohort study of 178 sixth-semester medical students at Karolinska Institutet, Stockholm, Sweden between the Spring of 2024 and the spring of 2025. Students completed questionnaires evaluating both platforms after experiencing them during their clinical rotation within rheumatology. A comparison between social artificial intelligence–enhanced robotic interface (blue color) and virtual interactive case (red color) is illustrated. The questionnaire item reads: Overall, working through this case was a worthwhile learning experience. Violin plots show score distributions; white boxes indicate the median and IQR, and numbers indicate mean scores. Q: question.

Students consistently rated SARI as superior to VIC across multiple domains of VP design that support CR training. Results from Likert-scale data are illustrated in Figures 1 and 2, Tables 1 and 2, with detailed response frequencies provided in Tables S1-S5 in Multimedia Appendix 2.

### Authenticity of Patient Encounters

SARI demonstrated higher authenticity ratings than VIC. Students experienced that they had to make decisions as a real-life clinician to a greater degree with SARI compared with VIC (median 4.0, IQR 3.0-4.0 vs 3.0, IQR 3.0-4.0; *W*=5306; *r*=0.34; *P*<.001) and felt with SARI more like a clinician being responsible for the care of the VP (median 4.0, IQR 3.0-4.0 vs 3.0, IQR 2.0-3.0; *W*=6936; *r*=0.58; *P*<.001). The overall theme score remained significantly greater for SARI (median 4.0, IQR 3.5-4.5 vs 3.0, IQR 2.5-3.5; *W*=9253; *r*=0.54; *P*<.001).

### Professional Approach During Consultation

Students reported significantly greater active engagement in CR processes when using SARI compared with VIC. This included more active engagement in gathering necessary clinical information (median 5.0, IQR 4.0-5.0 vs 4.0, IQR 3.0-5.0; *W*=4011; *r*=0.43; *P*<.001), revising their clinical impression as new information became available (median 4.0, 4.0-5.0 vs 4.0, IQR 4.0-5.0; *W*=1378; *r*=0.28; *P*<.001), creating structured patient summaries using medical terminology (median 4.0, IQR 3.0-5.0 vs 4.0, IQR 3.0-4.0; *W*=2146; *r*=0.43; *P*<.001), and actively considering findings that support or refute differential diagnoses (median 5.0, IQR 4.0-5.0 vs 4.0, IQR 4.0-5.0; *W*=868; *r*=0.27; *P*<.001). The overall theme score was significantly greater for SARI (median 4.5, IQR 4.0-4.8 vs 4, IQR 3.5-4.5; *W*=6717; *r*=0.54; *P*<.001).

### Coaching During Consultation

Students perceived no significant difference between platforms regarding case difficulty appropriateness for their training level (median 5.0, IQR 4.0-5.0 vs 5.0, IQR 4.0-5.0; *W*=248; *r*=0.11; *P*=.12). However, SARI was rated significantly better in facilitating helpful interactions that enhanced diagnostic reasoning (median 4.0, IQR 4.0-5.0 vs 4.0, IQR 3.3-5.0; *W*=1123; *r*=0.29; *P*<.001) and for system feedback that supported diagnostic reasoning (median 4.0, IQR 4.0-5.0 vs 4.0, IQR 3.0-5.0; *W*=1268; *r*=0.31; *P*<.001). The overall theme score favored SARI (median 4.3, IQR 4.0-4.7 vs 4.0, IQR 3.7-4.7; *W*=4092.5; *r*=0.32; *P*<.001).

### Learning Effect of Consultation

Students felt significantly better prepared for real clinical encounters after having used SARI compared with VIC. This included feeling better prepared to confirm the diagnosis and exclude differential diagnoses in real patients with similar complaints (median 4.0, IQR 4.0-5.0 vs 4.0, IQR 3.0-4.0; *W*=1491; *r*=0.35; *P*<.001) and feeling better prepared to provide care for real patients with similar conditions (median 4.0, IQR 4.0-5.0 vs 4.0, IQR 3.0-4.0; *W*=1515; *r*=0.39; *P*<.001). The overall theme score significantly favored SARI (median 4.0, IQR 4.0-5.0 vs 4.0, IQR 3.5-4.5; *W*=2589; *r*=0.42; *P*<.001).

### Overall Judgment

Students rated the interaction with SARI as a significantly more worthwhile learning experience compared with VIC (median 5.0, IQR 4.0-5.0 vs 4.0, IQR 4.0-5.0; *W*=2351; *r*=0.35; *P*<.001). However, both platforms received generally positive evaluations from the students.

### VP Platform Preference for CR Training

#### Visual Analogue Scale Ratings

**Figure 3:**
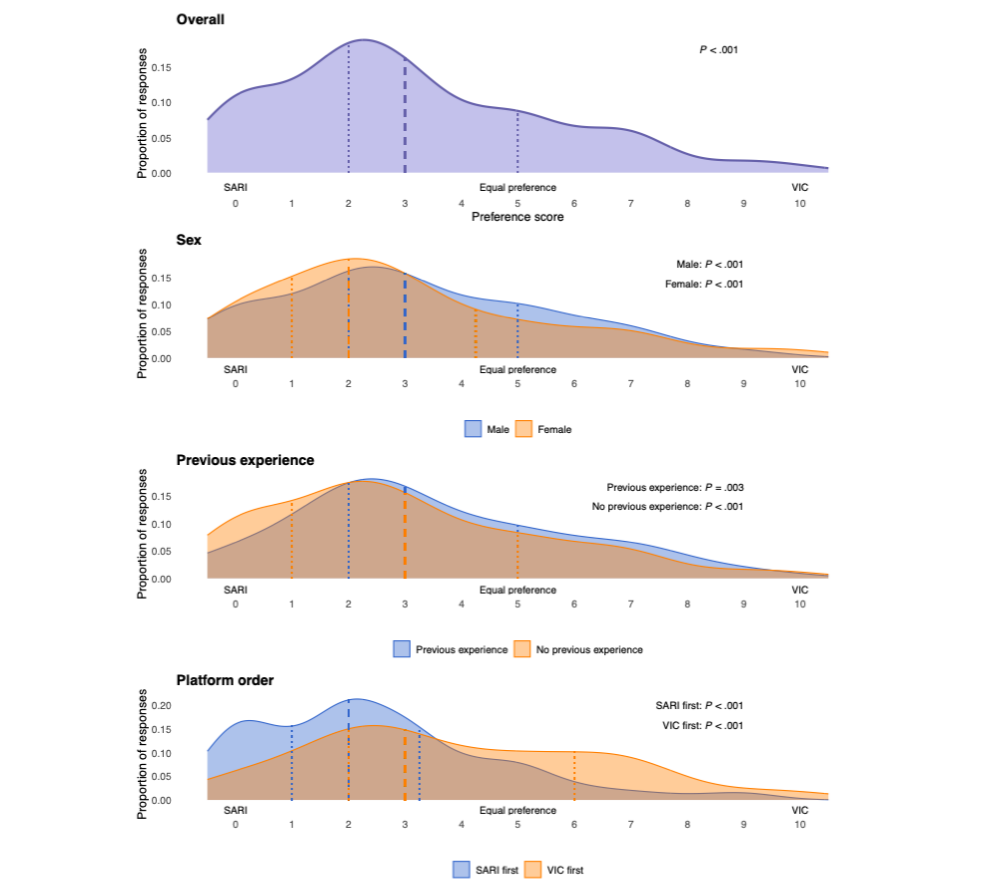
Density plots illustrating distributions of Visual Analogue Scale responses (0-10 scale; 0=total preference for SARI, 10=total preference for VIC, 5=equal preference of platforms) regarding virtual patient platform preference for CR training in an observational crossover cohort study of 178 sixth-semester medical students at Karolinska Institutet, Stockholm, Sweden, between the Spring of 2024 and the Spring of 2025. Results from Wilcoxon signed-rank tests are shown for comparisons of scores with a hypothetical score of 5 (equal preference of platforms) for each student. The panels show, from top to bottom, the overall distribution of responses in the entire cohort of students, overlayed distributions in women and men, overlayed distributions in students with and without prior experience of virtual patients, and overlayed distributions in subgroups of students starting with SARI or VIC. All students experienced both SARI and VIC during their clinical rotation within rheumatology, with platform order being determined by rotation scheduling. SARI: social artificial intelligence–enhanced robotic interface; VIC: virtual interactive case.

Students reported an overall strong preference for SARI over VIC for CR training (median 3.0, IQR 2.0-5.0; *W*=1604.5; *r*=0.60; *P*<.001). This preference pattern remained statistically significant across all subgroups of interest, that is, if students were female or male, if they had or did not have prior experience with VPs, and if they had started with SARI or VIC (Figure 3 and Tables S6-S9 in Multimedia Appendix 2).

#### Categorical Preference Responses

When asked to choose their preferred platform, students strongly favored SARI over VIC (72% vs 14%; OR 27.1, 95% CI 14.3-53.7; *P*<.001). The preference for SARI over equal preference was also statistically significant (72% vs 15%; OR 23.1, 95% CI 8.5-54.6; *P*<.001). When comparing SARI to VIC or equal preference combined, SARI remained strongly favored (72% vs 28%; OR 6.3, 95% CI 3.9-10.4; *P*<.001).

**Figure 4:**
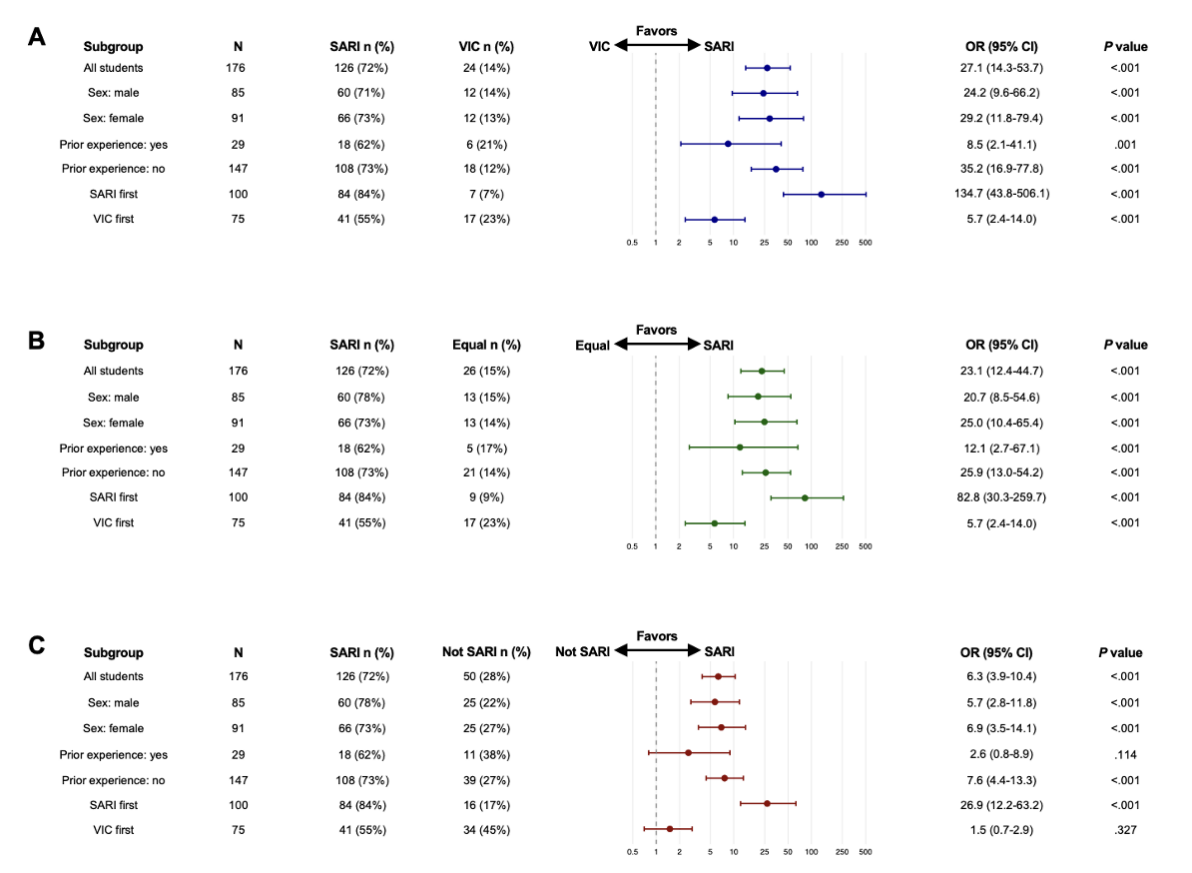
Forest plots illustrating results from Fisher exact test with Monte Carlo simulation (10,000 iterations) on categorical preferences for virtual patient platforms for clinical reasoning training in an observational crossover cohort study of 178 sixth-semester medical students at Karolinska Institutet, Stockholm, Sweden, between the Spring of 2024 and the Spring of 2025. Proportion of students preferring SARI versus comparator. Panel A shows comparisons between SARI and VIC (blue color). Panel B illustrates comparisons between SARI and equal preference (green color). Panel C shows comparisons between SARI and Not SARI (VIC or equal preference combined; red color). Circles denote ORs and whiskers denote 95% CIs on a logarithmic scale. All students experienced both SARI and VIC during their clinical rotation within rheumatology, with platform order being determined by rotation scheduling. Subgroups analyzed: overall cohort, female and male students, students with and without prior virtual patient experience, and students starting with SARI and students starting with VIC. OR: odds ratio; SARI: social artificial intelligence–enhanced robotic interface; VIC: virtual interactive case.

Similar patterns were observed across student subgroups in most comparisons. However, the preference difference did not reach statistical significance in the comparison between SARI and VIC or equal preference combined among students with prior VP experience (62% vs 38%; OR 2.6, 95% CI 0.8-8.9; *P*=.11) and those introduced to VIC first (55% vs 45%; OR 1.5, 95% CI 0.7-2.9; *P*=.33), although a numerical preference for SARI was still evident in both groups. Results are illustrated in Figure 4.

## Discussion

### Principal Findings

This observational crossover cohort study aimed to compare medical students’ experience of an AI-enhanced social robotic (SARI) versus a conventional computer-based VP platform (VIC), regarding the extent to which the design characteristics of the respective platform facilitate training of CR skills. Consistent with our hypothesis based on prior results from qualitative work [21,22], students perceived SARI as superior across all 5 domains of VP design, indicating that AI-enhanced social robotic VPs offer significant advantages over conventional computer-based platforms for CR training in medical education. Students also expressed a strong overall preference for SARI over VIC for CR training, suggesting that embodied AI systems may better support the cognitive processes underpinning effective CR skill development.

The effect sizes observed (*r* ranging from 0.27 to 0.60) represent moderate to large effects according to Cohen conventions, with the strongest effect seen for overall platform preference (*r*=0.60). While the Likert-scale ratings showed moderate advantages for design characteristics in SARI, the categorical preference data revealed much stronger student preference (OR 27.1), suggesting that the overall educational experience of SARI extends beyond individual distinct design elements captured by the Likert-scales of the study questionnaire.

Students perceived SARI as significantly more authentic than VIC in terms of feeling like they had to make decisions as real-life clinicians and feeling like the clinician was responsible for the care of the patient. Importantly, according to educational theory, authentic learning environments are crucial for effective CR development [16]. Our findings validate previous qualitative research from our group, which also demonstrated that SARI yields greater perceived authenticity compared with VIC [22]. The physical embodiment, combined with natural gaze behavior, facial expressions, and conversational AI, appears to create a more immersive clinical simulation that better prepares students for real patient encounters.

The superiority of SARI in promoting a professional approach during consultations suggests that social robotic embodiment facilitated more active cognitive engagement in CR processes. Students consistently reported being more actively engaged in gathering clinical information, revising their clinical impressions, creating structured patient summaries, and considering differential diagnoses when using SARI compared with VIC. This finding is particularly important, taking into consideration that effective VP platforms should specifically support active cognitive engagement rather than passive information processing [40,41]. The multimodal nature of SARI in combining visual, auditory, and conversational elements may activate multiple cognitive pathways that enhance learning retention and skill transfer [20-22].

The advantages of SARI in providing helpful interactions and feedback that promoted diagnostic reasoning suggest that AI-enhanced conversational interfaces can provide more adaptive and contextually appropriate educational support than traditional platforms. Students felt better prepared for real clinical encounters after having used SARI compared with VIC, indicating potential for improved skill transfer from digital to actual clinical practice. This finding addresses a critical challenge in medical education, which involves ensuring that VP experiences translate into improved real-world clinical competencies [18].

The superiority of SARI was generally robust across student subgroups. However, 2 notable exceptions warrant consideration. Students with prior VP experience showed only a numerical preference for SARI that did not reach statistical significance in the comparison with VIC or equal preference combined, suggesting that familiarity with VP technology may attenuate the perceived benefits of a new educational technology, such as the AI-enhanced social robotic interface introduced in this study. Additionally, students first introduced to VIC showed a smaller, nonsignificant preference margin for SARI, which may reflect first-impression or carryover effects, or both operating simultaneously. Such order effects are inherent limitations of crossover designs, where complete washout is neither feasible nor appropriate. Nevertheless, this finding is consistent with the earlier observation that familiarity may influence the perceived benefits of a new technology. Taken together, these findings indicate that while SARI generally offers advantages, perceived benefits may be influenced by students’ prior exposure to similar technologies, as well as the order in which technologies are encountered. While the crossover design mitigated learning effects, some degree of order effect likely remained, as complete elimination is inherently challenging.

Our results extend previous research on VPs in medical education by demonstrating quantifiable advantages of AI-enhanced social robotics. A recent systematic review identified the need for VP platforms that specifically target CR components such as problem presentation, hypothesis generation, and diagnostic justification [6]. Our findings suggest that AI-enhanced social robotic platforms may be well-suited for addressing these educational needs through their ability to provide dynamic, responsive interactions that mirror real clinical encounters more closely than traditional computer-based systems.

It is important to note that our results did not favor SARI over VIC consistently across all student subgroups. While this likely reflects limitations in statistical power, individual learner characteristics and contextual implementation factors may also impact students’ perceptions of the effectiveness of new educational modalities. Long-term studies examining sustained educational benefits would strengthen the evidence for AI-enhanced VP platforms. We would also like to emphasize that the 2 VP platforms incorporate different design features, and that the purpose of this study was to explore students’ perspectives on these design elements rather than evaluate the specific contributions of each platform to distinct aspects of CR, especially since no assessment of implemented CR skills was undertaken. We also acknowledge that our comparison between SARI and VIC concerns 2 specific platforms, and that results might differ if another AI-enhanced social robotic interface or a different conventional computer-based VP platform were used. Future research could further investigate the added value of platform-specific differences for CR training outcomes in implementation settings, for example, through examination-based evaluations.

### Limitations and Strengths

Several limitations warrant consideration when interpreting our results. First, our findings are based on students’ perceptions of VP design elements rather than objective measures of CR skill acquisition. Moreover, the internal consistency of the adapted questionnaire was not assessed in our study population. Future research that incorporates validated CR assessments and longitudinal evaluation of skill transfer to real clinical encounters is warranted [42]. While students consistently favored SARI, it is important to acknowledge that positive reception of novel technology does not always correlate with improved educational outcomes [43]. Novelty effects may have influenced students, as this was the first exposure to SARI for most participants, whereas computer-based platforms might already have been familiar to some.

Second, the single-center and discipline-specific design within rheumatology may limit the generalizability of the findings to other educational contexts or medical disciplines. Third, the use of English in case interaction rather than the students’ native language (Swedish for a vast majority) may have affected perceived authenticity and interaction quality, though this limitation likely affected both platforms similarly. Thus, we did not collect data on the students’ native language, precluding subgroup analysis based on language background to investigate how this might have influenced platform preferences.

Fourth, the use of a single LLM version (GPT-3.5-turbo) limits the generalizability of our findings across different AI models and their reproducibility over time. However, this choice ensured homogeneity across student groups in the present study. Given that LLM performance and behavior evolve with updates, newer models with enhanced capabilities may yield more reliable or nuanced results. Last, group size (pairs vs groups of 3) was not investigated, and we cannot exclude the possibility that group dynamics influenced individual perceptions, though students experienced both platforms in the same group configuration.

Our study also has several strengths. The observational crossover design allowed students to serve as their own controls, minimizing between-participant variability. We used a validated questionnaire specifically designed for VP design evaluation with emphasis on CR training. The study included a substantial sample (n=178) representing 42% of eligible students, which supports the generalizability of the findings within the target population. Furthermore, this is the first quantitative comparison of self-perceived VP design characteristics between an AI-enhanced social robotic VP platform and a conventional computer-based VP software for CR training within medical education.

### Implications

The findings of the present study have important implications for curricular development within medical education. AI-enhanced social robotic VP platforms may be considered superior alternatives to conventional computer-based systems, particularly for CR training. However, implementation decisions should also account for economic factors, technical infrastructure requirements, and faculty training needs. The integration of LLMs with social robotics represents a significant technological advancement that may justify investment despite potentially higher initial costs compared with traditional platforms. Over a longer term, the initial higher cost may mitigate costs from medical errors, through contribution to better preparation of students in advanced yet safe patient simulation environments.

### Conclusions

This study provides quantitative evidence that medical students may perceive AI-enhanced social robotic VP platforms as offering advantages in design characteristics over conventional computer-based platforms for CR training. The consistent superiority of SARI across multiple domains of VP design, combined with strong student preferences regardless of individual characteristics, suggests that embodied AI platforms represent a meaningful advancement in medical pedagogy technology. These findings support the integration of LLMs with social robotics as a promising approach for developing more effective VP simulations that better prepare medical students for real clinical encounters and warrant future research to examine objective CR performance outcomes and long-term learning retention. To our knowledge, this is the first quantitative head-to-head comparison of VP design characteristics between 2 technological approaches, one of which is a social robotic VP platform, thereby extending beyond prior qualitative and single-platform evaluations.

## Data Availability

The anonymized datasets generated and analyzed during this study are available from the corresponding author upon reasonable request. Access to data will be granted following appropriate ethical review and data sharing agreements and will require completion of a data transfer agreement and approval from the Swedish Ethical Review Authority, as per Swedish data protection regulations and the European General Data Protection Regulation.

## Appendix

Multimedia Appendix 1 Supplementary figures, including the VP platform evaluation questionnaire, example questions for structured history taking in rheumatology, an LLM-prompt example, and a schematic illustration of the social AI-enhanced robotic interface (SARI) workflow.

Multimedia Appendix 2 Supplementary tables, including frequencies of responses to the VP evaluation questionnaire, and Wilcoxon signed rank test results from VAS data on student preference of VP platforms.

Multimedia Appendix 3 Strengthening the Reporting of Observational Studies in Epidemiology (STROBE) checklist.

Multimedia Appendix 4 Guideline for reporting evidence-based practice educational interventions and teaching (GREET) checklist.

## Abbreviations

AI: artificial intelligence
CR: clinical reasoning
GREET: Guideline for Reporting Evidence-Based Practice Educational Interventions and Teaching
KI: Karolinska Institutet
LLM: large language model
OR: odds ratio
SARI: social artificial intelligence–enhanced robotic interface
STROBE: Strengthening the Reporting of Observational Studies in Epidemiology
VAS: Visual Analogue Scale
VIC: virtual interactive case
VP: virtual patient

## References

[1] Ellaway R, Candler C, Greene P, Smothers V (2006). An Architectural Model for Medbiquitous Virtual Patients.

[2] Consorti F, Mancuso R, Nocioni M, Piccolo A (2012). Efficacy of virtual patients in medical education: a meta-analysis of randomized studies. Comput Educ.

[3] Yardley S, Teunissen PW, Dornan T (2012). Experiential learning: transforming theory into practice. Med Teach.

[4] Cook DA, Triola MM (2009). Virtual patients: a critical literature review and proposed next steps. Med Educ.

[5] Ellaway R (2006). Weaving the 'e's together. Med Teach.

[6] Plackett R, Kassianos AP, Mylan S, Kambouri M, Raine R, Sheringham J (2022). The effectiveness of using virtual patient educational tools to improve medical students' clinical reasoning skills: a systematic review. BMC Med Educ.

[7] Fąferek J, Cariou P, Hege I, Mayer A, Morin L, Rodriguez-Molina D, Sousa-Pinto B, Kononowicz AA (2024). Integrating virtual patients into undergraduate health professions curricula: a framework synthesis of stakeholders' opinions based on a systematic literature review. BMC Med Educ.

[8] Edelbring S, Dastmalchi M, Hult H, Lundberg IE, Dahlgren LO (2011). Experiencing virtual patients in clinical learning: a phenomenological study. Adv Health Sci Educ Theory Pract.

[9] Huwendiek S, Reichert F, Bosse H, de Leng BA, van der Vleuten CP M, Haag M, Hoffmann GF, Tönshoff B (2009). Design principles for virtual patients: a focus group study among students. Med Educ.

[10] Young M, Thomas A, Lubarsky S, Ballard T, Gordon D, Gruppen L, Holmboe E, Ratcliffe T, Rencic J, Schuwirth L, Durning SJ (2018). Drawing Boundaries: the difficulty in defining clinical reasoning. Acad Med.

[11] Eva KW (2005). What every teacher needs to know about clinical reasoning. Med Educ.

[12] Higgs J, Jensen GM, Loftus S, Trede FV, Grace S (2024). Clinical Reasoning in the Health Professions E-Book.

[13] Graber ML, Franklin N, Gordon R (2005). Diagnostic error in internal medicine. Arch Intern Med.

[14] Parodis I, Andersson L, Durning SJ, Hege I, Knez J, Kononowicz AA, Lidskog M, Petreski T, Szopa M, Edelbring S (2021). Clinical reasoning needs to be explicitly addressed in health professions curricula: recommendations from a european consortium. Int J Environ Res Public Health.

[15] Daniel M, Rencic J, Durning S, Holmboe E, Santen S, Lang V, Ratcliffe T, Gordon D, Heist B, Lubarsky S, Estrada CA, Ballard T, Artino AR, Sergio Da Silva A, Cleary T, Stojan J, Gruppen LD (2019). Clinical reasoning assessment methods: a scoping review and practical guidance. Acad Med.

[16] Cook DA, Erwin PJ, Triola MM (2010). Computerized virtual patients in health professions education: a systematic review and meta-analysis. Acad Med.

[17] Kononowicz AA, Woodham LA, Edelbring S, Stathakarou N, Davies D, Saxena N, Tudor Car L, Carlstedt-Duke J, Car J, Zary N (2019). Virtual patient simulations in health professions education: systematic review and meta-analysis by the digital health education collaboration. J Med Internet Res.

[18] Jay R, Sandars J, Patel R, Leonardi-Bee J, Ackbarally Y, Bandyopadhyay S, Faraj D, O'Hanlon M, Brown J, Wilson E (2025). The use of virtual patients to provide feedback on clinical reasoning: a systematic review. Acad Med.

[19] Gordon M, Daniel M, Ajiboye A, Uraiby H, Xu NY, Bartlett R, Hanson J, Haas M, Spadafore M, Grafton-Clarke C, Gasiea RY, Michie C, Corral J, Kwan B, Dolmans D, Thammasitboon S (2024). A scoping review of artificial intelligence in medical education: BEME guide no. 84. Med Teach.

[20] Borg A, Parodis I, Skantze G (2024). Creating virtual patients using robots and large language models: a preliminary study with medical students.

[21] Borg A, Georg C, Jobs B, Huss V, Waldenlind K, Ruiz M, Edelbring S, Skantze G, Parodis I (2025). Virtual patient simulations using social robotics combined with large language models for clinical reasoning training in medical education: mixed methods study. J Med Internet Res.

[22] Borg A, Jobs B, Huss V, Gentline C, Espinosa F, Ruiz M, Edelbring S, Georg C, Skantze G, Parodis I (2024). Enhancing clinical reasoning skills for medical students: a qualitative comparison of LLM-powered social robotic versus computer-based virtual patients within rheumatology. Rheumatol Int.

[23] Abd-Alrazaq A, AlSaad R, Alhuwail D, Ahmed A, Healy PM, Latifi S, Aziz S, Damseh R, Alabed Alrazak S, Sheikh J (2023). Large language models in medical education: opportunities, challenges, and future directions. JMIR Med Educ.

[24] Zhui L, Yhap N, Liping L, Zhengjie W, Zhonghao X, Xiaoshu Y, Hong C, Xuexiu L, Wei R (2024). Impact of large language models on medical education and teaching adaptations. JMIR Med Inform.

[25] Vrdoljak J, Boban Z, Vilović M, Kumrić M, Božić J (2025). A review of large language models in medical education, clinical decision support, and healthcare administration. Healthcare (Basel).

[26] Cohen A, Sur M, Weisse M, Moffett K, Lancaster J, Saggio R, Singhal G, Thammasitboon S (2020). Teaching diagnostic reasoning to faculty using an assessment for learning tool: training the trainer. MedEdPORTAL.

[27] Gruppen LD (2017). Clinical reasoning: defining it, teaching it, assessing it, studying it. West J Emerg Med.

[28] (2024). Virtual Interactive Case System.

[29] von Elm E, Altman DG, Egger M, Pocock SJ, Gøtzsche PC, Vandenbroucke JP, STROBE Initiative (2007). The strengthening the reporting of observational studies in epidemiology (STROBE) statement: guidelines for reporting observational studies. Lancet.

[30] Phillips AC, Lewis LK, McEvoy MP, Galipeau J, Glasziou P, Moher D, Tilson JK, Williams MT (2016). Development and validation of the guideline for reporting evidence-based practice educational interventions and teaching (GREET). BMC Med Educ.

[31] Huwendiek S, De Leng BA, Kononowicz AA, Kunzmann R, Muijtjens AMM, Van Der Vleuten CPM, Hoffmann GF, Tönshoff B, Dolmans DHJM (2015). Exploring the validity and reliability of a questionnaire for evaluating virtual patient design with a special emphasis on fostering clinical reasoning. Med Teach.

[32] Posel N, Fleiszer D, Shore BM (2009). 12 Tips: guidelines for authoring virtual patient cases. Med Teach.

[33] Posel N, Mcgee JB, Fleiszer DM (2015). Twelve tips to support the development of clinical reasoning skills using virtual patient cases. Med Teach.

[34] Edelbring S, Parodis I, Lundberg IE (2018). Increasing reasoning awareness: video analysis of students' two-party virtual patient interactions. JMIR Med Educ.

[35] Moubayed S AI, Beskow J, Skantze G, Granström B (2012). Furhat: a back-projected human-like robot head for multiparty human-machine interaction.

[36] Mishra C, Offrede T, Fuchs S, Mooshammer C, Skantze G (2023). Does a robot's gaze aversion affect human gaze aversion?. Front Robot AI.

[37] (2022). ChatGPT:optimizing language models for dialogue. OpenAI.

[38] Irfan B, Kuoppamäki SM, Hosseini A, Skantze G (2023). Between reality and delusion: challenges of applying large language models to companion robots for open-domain dialogues with older adults. Auton Robot.

[39] Huwendiek S, De leng BA, Zary N, Fischer MR, Ruiz JG, Ellaway R (2009). Towards a typology of virtual patients. Med Teach.

[40] Hege I, Kononowicz AA, Adler M (2017). A clinical reasoning tool for virtual patients: design-based research study. JMIR Med Educ.

[41] Gordon D, Rencic JJ, Lang VJ, Thomas A, Young M, Durning SJ (2022). Advancing the assessment of clinical reasoning across the health professions: definitional and methodologic recommendations. Perspect Med Educ.

[42] Siegelman J, Bernstein L, Goedken J, Lewin L, Schneider J, Ward M, Stoddard H (2024). Assessment of clinical reasoning during a high stakes medical student OSCE. Perspect Med Educ.

[43] Vieriu AM, Petrea G (2025). The impact of artificial intelligence (AI) on students’ academic development. Educ Sci.

