# Supplementary material for: AI-Enhanced Social Robotic Versus Computer-Based Virtual Patients for Clinical Reasoning Training in Medical Education: Observational Crossover Cohort Study
Source: J Med Internet Res. 2025 Nov 27;27:e82541. doi: 10.2196/82541 (PMC12699248; doi:10.2196/82541)

**Figure S1.** Questionnaire to evaluate virtual patient platform design with an emphasis on clinical reasoning.



# Clinical reasoning evaluation

Please complete the survey below.

Thank you!

---

Identifier ______________________________

---

Student questionnaire concerning the learning and clinical reasoning experiences with virtual patients

---

About this questionnaire

This questionnaire is for students to evaluate their experiences with virtual patients, comparing a large language model-driven social robot (Furhat) to a computer-based platform (VIC) , focusing on the development of clinical reasoning skills.

This questionnaire contains 14 items clustered into seven subsets. This instrument can be repeatedly administered to elicit student's experiences immediately following each workshop or 'play' of a virtual patient.

Please respond using the following 5-point scale:

1) Strongly disagree

2) Disagree

3) Neutral

4) Agree

5) Strongly agree

6) Not applicable

Please indicate briefly the reason(s) for your response for each question (optional).

## Participant information

| | |
|---|---|
| Legal gender | ○ Male<br>○ Female |
| Age | ______________________________<br>(Please provide your age as a number (For example: 27)) |
| Username when using the social robot<br><br>Remember this so that you can return to your interactions and generate feedback | ______________________________<br>(StudentX) |
| Personal number | ______________________________<br>(yyyymmdd-xxxx) |

| | |
|---|---|
| Which health profession education programme are you a student within? | ◯ Medical programme<br>◯ Nursing programme<br>◯ Physiotherapy programme<br>◯ Occupational therapy programme |
| Which term of studies are you currently attending? | ◯ 1<br>◯ 2<br>◯ 3<br>◯ 4<br>◯ 5<br>◯ 6<br>◯ 7<br>◯ 8<br>◯ 9<br>◯ 10<br>◯ 11<br>◯ 12 |
| Do you have any previous experience of virtual patient simulations? | ◯ Yes<br>◯ No |
| Please specify | ________________________________ |
| The platform that I was introduced to first was | ◯ The social robot (Furhat)<br>◯ The computer-based platform (VIC) |

I completed the virtual patient cases

Social robot (Furhat) Computer-based platform (VIC)

______ ______

Date of case completion

Fill in the same date if uncertain

Social robot Computer-based platform

______ ______

## Authenticity of patient encounter and the consultation

While working on this case, I felt I had to make the same decisions a clinican would make in real life.

Social robot Computer-based platform

______ ______

Please indicate briefly the reason(s) for your response to the question above (optional)

________________________________

While working on this case, I felt I were the clinician caring for this patient

Social robot Computer-based platform

______

______

Please indicate briefly the reason(s) for your response to the question above (optional)

______________________

## Professional approach in the consultation

While working through this case, I was actively engaged in gathering the information (e.g., history questions, physical exams, lab tests) I needed, to characterise the patient's problem

Social robot Computer-based platform

______ ______

Please indicate briefly the reason(s) for your response to the question above (optional)

______________________

While working through this case, I was actively engaged in revising my initial image of the patient's problem as new information became available

Social robot Computer-based platform

______ ______

Please indicate briefly the reason(s) for your response to the question above (optional)

______________________

While working through this case, I was actively engaged in creating a short summary of the patient's problem using medical terms

Social robot Computer-based platform

______ ______

Please indicate briefly the reason(s) for your response to the question above (optional)

______________________

While working through this case, I was actively engaged in thinking about which findings supported or refuted each diagnosis in my differential diagnosis

Social robot Computer-based platform

______ ______

Please indicate briefly the reason(s) for your response to the question above (optional)

______________________

## Coaching during consultation

I felt that the case was at the appropriate level of difficulty for my level of training

Social robot Computer-based platform

______ ______

Please indicate briefly the reason(s) for your response to the question above (optional)

________________________________________

The questions I was asked while working through this case were helpful in enhancing my diagnostic reasoning in this case

Social robot Computer-based platform

______ ______

Please indicate briefly the reason(s) for your response to the question above (optional)

________________________________________

The feedback I received was helpful in enhancing my diagnostic reasoning in this case

Social robot Computer-based platform

______ ______

Please indicate briefly the reason(s) for your response to the question above (optional)

________________________________________

## Learning effect of consultation

After completing this case, I feel better prepared to confirm a diagnosis and exclude differential diagnoses in a real life patient with this complaint

Social robot Computer-based platform

______ ______

Please indicate briefly the reason(s) for your response to the question above (optional)

________________________________________

After completing this case, I feel better prepared to care for a real life patient with this complaint

Social robot Computer-based platform

______ ______

Please indicate briefly the reason(s) for your response to the question above (optional)

________________________________________

## Overall judgment of case workup

Overall, working through this case was a worthwile learning experience

Social robot Computer-based platform

______ ______

Please indicate briefly the reason(s) for your response to the question above (optional)

________________________________________

## Open-ended questions

Special strenghts of the platform

Social robot Computer-based platform

______ ______

Special weaknesses of the platform

Social robot Computer-based platform

______ ______

## Preference and enjoyability

On a scale of 0-10, where 0 is a total preference of the social robot and 10 is a total preference of the computer-based platform, how would you grade your preference of the virtual patient platforms compared to each other for acquirement of clinical reasoning skills?

Social robot — Equally preferred — Computer-based platform

*(Place a mark on the scale above)*

Please specify (optional)

________________________________________

Overall, which of the platforms is preferable to you for acquirement of clinical reasoning skills?

- ◯ Social robot
- ◯ Computer-based platform
- ◯ Equally preferred

On a scale of 0-10, where 0 is a total preference of the social robot and 10 is a total preference of the computer-based platform, how would you grade your preference of the virtual patient platforms compared to each other in relation to self-experienced empathy during the patient encounter?

Social robot — Equally preferred — Computer-based platform

*(Place a mark on the scale above)*

Please specify (optional)

________________________________________

Overall, which of the platforms is preferable to you in relation to self-experienced empathy during the patient encounter?

- ◯ Social robot
- ◯ Computer-based platform
- ◯ Equally preferred

| | |
|---|---|
| On a scale of 0-10, where 0 is a total preference of the social robot and 10 is a total preference of the computer-based platform, how would you grade your preference of the virtual patient platforms compared to each other in relation to gathering medical history from a patient? | Social robot — Equally preferred — Computer-based platform<br>*(Place a mark on the scale above)* |
| Please specify (optional) | ________________________ |
| Overall, which of the platforms is preferable to you in relation to gathering medical history from a patient? | ◯ Social robot<br>◯ Computer-based platform<br>◯ Equally preferred |

How enjoyable would you grade your total experience with the platforms?

Social robot Computer-based platform

______ ______

Any additional comments? (optional)

________________________

**Figure S2.** Structured history taking in rheumatology with example questions.

**Reason for consultation**

- What brings you here today?
- What do you hope to get out of the visit today?
- What do you think about your symptoms yourself?
- Do you have any particular concerns or worries about your symptoms?

**Present illness**

**Systemic symptoms**

- Have you experienced fever, low-grade pyrexia, or feverish feelings?
- Have you had any unintentional weight loss? If yes, how much and over what period?
- Have you experienced night sweats?
- Do you feel generally unwell/do you have a general feeling of malaise?
- Have you had any clots (thromboembolic events)?

**Musculoskeletal symptoms**

- Do you have muscle pain?
- How would you describe the pain?
- Where is the pain located?
- Does the pain radiate anywhere?
- Is the pain symmetrical?
- Do you have claudication (pain on exertion) in your arms?
- Do you have joint pain?
- Where is the joint pain located (number, localisation)?
- Is it symmetrical?
- Do you have swollen joints?
- Do you have any sausage-like swelling in fingers or toes (dactylitis)?
- Do you have pain where muscles/tendons attach (enthesitis)?
- Do you have back pain?
- When did it start? At what age?
- Does it have inflammatory characteristics?
- How does the pain vary throughout the day?
- How is it affected by rest?
- Does it improve with movement or exercise?
- Do you experience morning stiffness?
- How long does it last?
- Do you have muscle weakness?
- Is it proximal or distal?
- How do your symptoms vary throughout the day?
- Do you have difficulty performing daily activities or do you need support?

## Other organ systems

### Head/face

- Do you have any new-onset headache?
- Have you experienced any new and severe hair loss?
- Do you have tenderness over the temporal artery?
- Do you have jaw claudication (pain when chewing)?
- Do you have recurrent sinusitis?
- Do you have nosebleeds or nasal crusts?
- Have you experienced ulcers or blisters in your mouth?
- Do you have reduced hearing?
- Do you have difficulty swallowing?
- Have you experienced symptoms from the nervous system (e.g., CNS: headache, seizures, et cetera)?

### Eyes

- Have you experienced visual changes?
- Do you have visual loss?
- Do you have double vision?
- Do you have blurred vision?
- Do you have eye problems such as red eye or light sensitivity?
- Have you experienced dryness in eyes and/or mouth?

### Respiratory system

- Do you have lung symptoms?
- Do you have a cough?
- Have you coughed up blood (haemoptysis)?
- Do you have shortness of breath?

### Skin

- Do you have any skin rashes?
- Have you been examined by a dermatologist?
- Do you have psoriasis?
- Do you have specific rashes (Gottron’s sign, shawl sign)?
- Do you have Raynaud’s phenomenon?
- Mono-, bi- or triphasic?
- For how long?
- Do you experience photosensitivity?

**Abdomen/urinary tract**

- Do you have any gastrointestinal symptoms?
- Do you have any kidney or urinary tract problems?
- Have you noticed any changes in bowel or urinary habits?

**Reproductive organs**

- Ulcers/blisters in the genital area?
- Miscarriages?
- How many?
- Early or late in pregnancy?

**Past medical history**

- Do you have any previously treated conditions?
- Have you undergone any operations?

**Family history**

- Do you have any family history of:
    - Rheumatic diseases?
    - Muscle diseases?
    - Psoriasis?
    - Spondylitis?
    - Cancer?

**Lifestyle**

- Do you smoke? Quantify
- Do you drink alcohol? Quantify
- How physically active are you? Quantify
- What are your eating habits like?
- How do you sleep?

**Social circumstances**

- Who are your closest relatives?

- How do you live?
- What do you work as?

**Current medications**

- What medications are you currently taking?
- Have you taken NSAIDs or other analgesics?
- How effective are your medications?

**Allergies**

- Do you have any allergies?

**Figure S3.** Example of prompt used for the social AI-enhanced robotic interface (SARI).

Mikael is about to meet his physician for the first time. Mikael is a 68 years old man who made an appointment a few days back. Apart from the age and sex of the patient, the only information the physician has is that Mikael has sought care because of "ache in the body".

**The following is some information about Mikael's condition [shortened]:**

- He has always been healthy and has not felt any pain similar to that he is seeking for now.
- Sometimes, he experiences back pain, but it has never been present for that long and it has not had the same character.
- In Mikael's medical charts, it is stated that his blood lipids are above normal.
- Mikael has been advised to eat healthier to avoid developing diabetes and lower his blood lipids.
- Mikael has had a stable blood pressure since he has been on anti-hypertensive treatment.

**The following is a dialogue between Mikael and his physician:**

- Mikael: Hi doctor.
- Physician: Hello Mikael, my name is Morgan.
- Mikael: Nice to meet you, Morgan. Thanks for seeing me.
- Physician: Of course! Could you describe why you are here today?

**Write the next line that Mikael would say.**

**Figure S4.** Schematic illustration of the social AI-enhanced robotic interface (SARI) workflow.

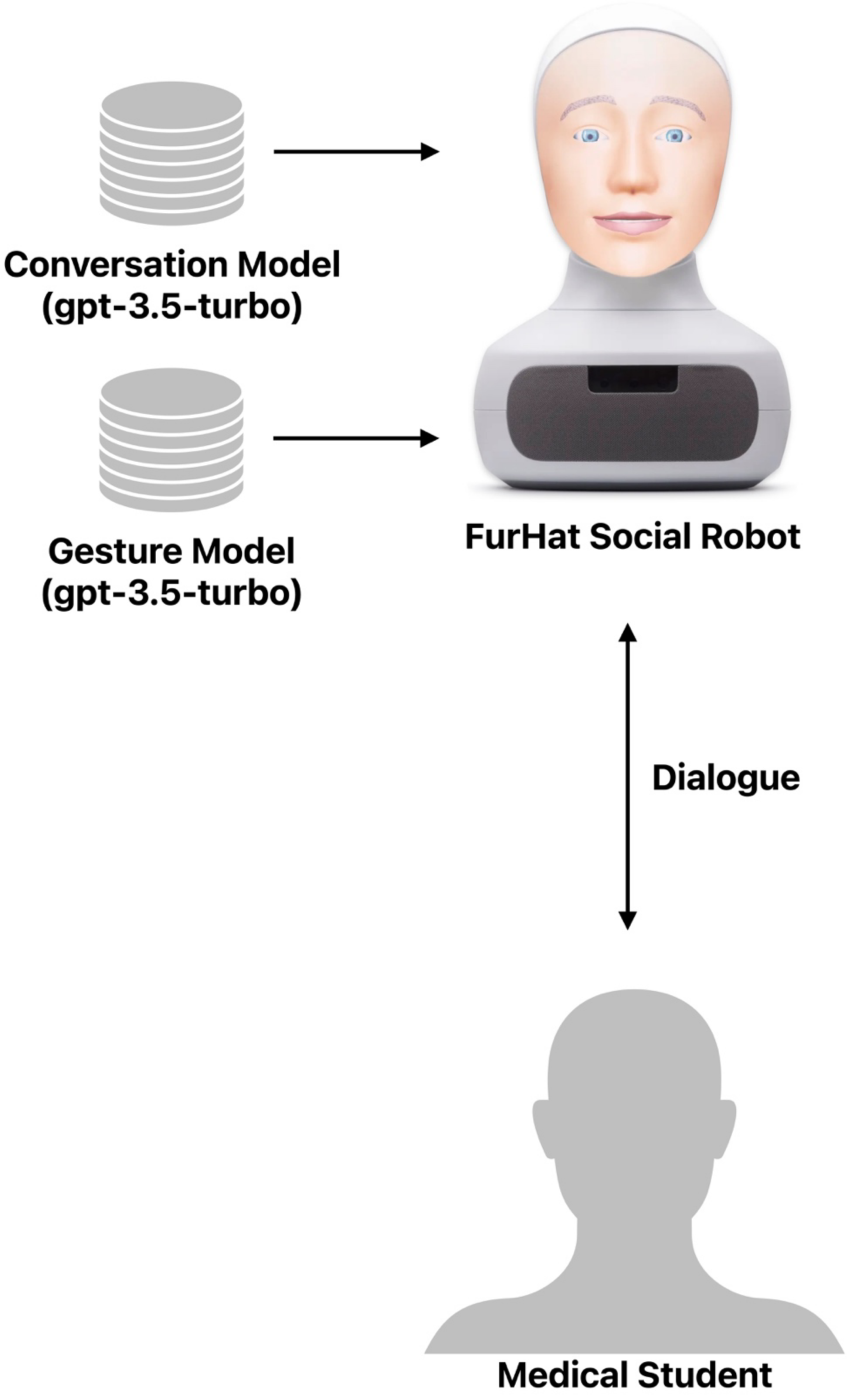

Supplement: Multimedia Appendix 1 [file jmir_v27i1e82541_app1.pdf]
