# Supplementary material for: AI-Enhanced Social Robotic Versus Computer-Based Virtual Patients for Clinical Reasoning Training in Medical Education: Observational Crossover Cohort Study
Source: J Med Internet Res. 2025 Nov 27;27:e82541. doi: 10.2196/82541 (PMC12699248; doi:10.2196/82541)
Supplement: Multimedia Appendix 2 [file jmir_v27i1e82541_app2.pdf]

Table S1. Responses to the clinical reasoning questionnaire within the theme “authenticity of patient encounter”.

**Authenticity of patient encounter in the consultation**		
While working on this case, I felt I had to make the same decisions a doctor would make in real life.		
**Response**	**SARI**	**VIC**
Strongly disagree	5 (2.9%)	4 (2.3%)
Disagree	16 (9.2%)	29 (16.7%)
Neutral	25 (14.4%)	57 (32.8%)
Agree	89 (51.1%)	70 (40.2%)
Strongly agree	38 (21.8%)	13 (7.5%)
Not applicable	1 (0.6%)	1 (0.6%)
While working on this case, I felt I were the doctor caring for this patient.		
**Response**	**SARI**	**VIC**
Strongly disagree	13 (7.5%)	24 (13.8%)
Disagree	11 (6.3%)	51 (29.3%)
Neutral	37 (21.3%)	60 (34.5%)
Agree	78 (44.8%)	33 (19.0%)
Strongly agree	35 (20.1%)	5 (2.9%)
Not applicable	0 (0.0%)	1 (0.6%)

Data are presented as numbers (percentage).

SARI: social AI-enhanced robotic interface; VIC: virtual interactive case simulator.

Table S2. Responses to the clinical reasoning questionnaire within the theme "professional approach in the consultation".

**Professional approach in the consultation**		
While working through this case, I was actively engaged in gathering the information (e.g., history questions, physical exams, lab tests) I needed, to characterise the patient's problem.		
**Response**	**SARI**	**VIC**
Strongly disagree	0 (0.0%)	3 (1.7%)
Disagree	5 (2.9%)	13 (7.5%)
Neutral	13 (7.5%)	36 (20.7%)
Agree	61 (35.1%)	75 (43.1%)
Strongly agree	95 (54.6%)	45 (25.9%)
Not applicable	0 (0.0%)	2 (1.1%)
While working through this case, I was actively engaged in revising my initial image of the patient's problem as new information became available.		
**Response**	**SARI**	**VIC**
Strongly disagree	3 (1.7%)	2 (1.1%)
Disagree	4 (2.3%)	5 (2.9%)
Neutral	10 (5.7%)	27 (15.5%)
Agree	75 (43.1%)	83 (47.7%)
Strongly agree	82 (47.1%)	56 (32.2%)
Not applicable	0 (0.0%)	1 (0.6%)
While working through this case, I was actively engaged in creating a short summary of the patient's problem using medical terms.		
**Response**	**SARI**	**VIC**
Strongly disagree	6 (3.4%)	11 (6.3%)
Disagree	6 (3.4%)	22 (12.6%)
Neutral	33 (19.0%)	40 (23.0%)
Agree	70 (40.2%)	59 (33.9%)
Strongly agree	57 (32.8%)	37 (21.3%)
Not applicable	2 (1.1%)	5 (2.9%)
While working through this case, I was actively engaged in thinking about which findings supported or refuted each diagnosis in my differential diagnosis.		
**Response**	**SARI**	**VIC**
Strongly disagree	0 (0.0%)	0 (0.0%)
Disagree	2 (1.2%)	2 (1.2%)
Neutral	4 (2.3%)	16 (9.3%)
Agree	60 (34.9%)	71 (41.3%)
Strongly agree	105 (61.0%)	82 (47.7%)
Not applicable	1 (0.6%)	1 (0.6%)

Data are presented as numbers (percentage).

SARI: Social AI-enhanced Robotic Interface; VIC: Virtual Interactive Case simulator.

Table S3. Responses to the clinical reasoning questionnaire within the theme “coaching during consultation”.

**Coaching during consultation**		
I felt that the case was at the appropriate level of difficulty for my level of training.		
**Response**	**SARI**	**VIC**
Strongly disagree	2 (1.1%)	2 (1.1%)
Disagree	5 (2.9%)	7 (4.0%)
Neutral	9 (5.2%)	12 (6.9%)
Agree	61 (35.1%)	59 (33.9%)
Strongly agree	97 (55.7%)	94 (54.0%)
Not applicable	0 (0.0%)	0 (0.0%)
The questions I was asked while working through this case were helpful in enhancing my diagnostic reasoning in this case.		
**Response**	**SARI**	**VIC**
Strongly disagree	2 (1.2%)	1 (0.6%)
Disagree	3 (1.7%)	9 (5.2%)
Neutral	18 (10.5%)	30 (17.4%)
Agree	62 (36.0%)	62 (36.0%)
Strongly agree	77 (44.8%)	57 (33.1%)
Not applicable	10 (5.8%)	13 (7.6%)
The feedback I received was helpful in enhancing my diagnostic reasoning in this case.		
**Response**	**SARI**	**VIC**
Strongly disagree	1 (0.6%)	10 (5.7%)
Disagree	4 (2.3%)	12 (6.9%)
Neutral	28 (16.0%)	39 (22.3%)
Agree	66 (37.7%)	48 (27.4%)
Strongly agree	59 (33.7%)	49 (28.0%)
Not applicable	17 (9.7%)	17 (9.7%)

Data are presented as numbers (percentage).

SARI: Social AI-enhanced Robotic Interface; VIC: Virtual Interactive Case simulator.

Table S4. Responses to the clinical reasoning questionnaire within the theme "learning effect of consultation".

Learning effect of consultation		
After completing this case, I feel better prepared to confirm a diagnosis and exclude differential diagnoses in a real-life patient with this complaint.		
**Response**	**SARI**	**VIC**
Strongly disagree	2 (1.1%)	2 (1.1%)
Disagree	6 (3.4%)	11 (6.3%)
Neutral	20 (11.4%)	37 (21.1%)
Agree	82 (46.9%)	87 (49.7%)
Strongly agree	65 (37.1%)	38 (21.7%)
Not applicable	0 (0.0%)	0 (0.0%)
After completing this case, I feel better prepared to care for a real-life patient with this complaint.		
**Response**	**SARI**	**VIC**
Strongly disagree	2 (1.1%)	2 (1.1%)
Disagree	7 (4.0%)	14 (8.0%)
Neutral	21 (12.1%)	38 (21.8%)
Agree	82 (47.1%)	87 (50.0%)
Strongly agree	62 (35.6%)	32 (18.4%)
Not applicable	0 (0.0%)	0 (0.6%)

Data are presented as numbers (percentage).

SARI: Social AI-enhanced Robotic Interface; VIC: Virtual Interactive Case simulator.

Table S5. Responses to the clinical reasoning questionnaire within the theme "overall judgment of case workup".

**Overall judgment of case workup**		
Overall, working through this case was a worthwile learning experience.		
**Response**	**SARI**	**VIC**
Strongly disagree	1 (0.6%)	1 (0.6%)
Disagree	4 (2.3%)	8 (4.6%)
Neutral	8 (4.6%)	19 (10.9%)
Agree	42 (24.0%)	72 (41.1%)
Strongly agree	120 (68.6%)	75 (42.9%)
Not applicable	0 (0.0%)	0 (0.0%)

Data are presented as numbers (percentage).

SARI: Social AI-enhanced Robotic Interface; VIC: Virtual Interactive Case simulator.

Table S6. Overall Wilcoxon signed-rank test results from VAS data on student preference of VP platforms.

Statistical metrics	Value
N	177
Median (IQR)	3.0 (2.0–5.0)
Mean (SD)	3.12 (± 2.4)
Positive ranks (scores > 5)	30
Negative ranks (scores < 5)	128
Ties (scores = 5)	19
Wilcoxon W	1604.5
Z	8.2
p value	<0.001
Effect size (r)	0.6

IQR: interquartile range; SD: standard deviation; VAS: visual analogue scale; VP: virtual patient.

SARI: Social AI-enhanced Robotic Interface; VIC: Virtual Interactive Case simulator.

Table S7. Wilcoxon signed-rank test results from VAS data on student preference of VP platforms based on student subgroups stratified by sex.

Statistic	Males	Females
N	85	92
Median (IQR)	3.0 (2.0–5.0)	2.0 (1.0–4.3)
Mean (SD)	3.2 (± 2.4)	3.0 (± 2.5)
Positive ranks (scores > 5)	15	15
Negative ranks (scores < 5)	59	69
Ties (scores = 5)	11	8
Wilcoxon W	355.0	464.0
Z	5.6	5.9
P-value	<0.001	<0.001
Effect size (r)	0.61	0.62

IQR: interquartile range; SD: standard deviation; VAS: visual analogue scale; VP: virtual patient.

SARI: Social AI-enhanced Robotic Interface; VIC: Virtual Interactive Case simulator.

Table S8. Wilcoxon signed-rank test results from VAS data on student preference of VP platforms based on student subgroups stratified based on previous VP experience.

Statistic	With prior VP experience	No prior VP experience
N	29	148
Median (IQR)	3.0 (2.0–5.0)	3.0 (1.0–5.0)
Mean (SD)	3.5 (± 2.3)	3.1 (± 2.4)
Positive ranks (scores > 5)	5	25
Negative ranks (scores < 5)	19	109
Ties (scores = 5)	5	14
Wilcoxon W	46.5	1118.0
Z	3.0	7.6
P-value	0.003	<0.001
Effect size (r)	0.6	0.6

IQR: interquartile range; SD: standard deviation; VAS: visual analogue scale; VP: virtual patient.

SARI: Social AI-enhanced Robotic Interface; VIC: Virtual Interactive Case simulator.

Table S9. Wilcoxon signed-rank test results from VAS data on student preference of VP platforms based on student subgroups stratified based on platform order.

Statistic	SARI first	VIC first
N	100	76
Median (IQR)	2.0 (1.0–3.3)	3.0 (2.0–6.0)
Mean (SD)	2.5 (± 2.1)	3.9 (± 2.5)
Positive ranks (scores > 5)	8	21
Negative ranks (scores < 5)	83	45
Ties (scores = 5)	9	10
Wilcoxon W	232.0	536.5
Z	7.4	3.7
P-value	<0.001	<0.001
Effect size (r)	0.74	0.42

IQR: interquartile range; SD: standard deviation; VAS: visual analogue scale; VP: virtual patient.

SARI: Social AI-enhanced Robotic Interface; VIC: Virtual Interactive Case simulator.
