# Supplementary material for: AI-Enhanced Social Robotic Versus Computer-Based Virtual Patients for Clinical Reasoning Training in Medical Education: Observational Crossover Cohort Study
Source: J Med Internet Res. 2025 Nov 27;27:e82541. doi: 10.2196/82541 (PMC12699248; doi:10.2196/82541)
Supplement: Multimedia Appendix 3 [file jmir_v27i1e82541_app3.pdf]

STROBE Statement—checklist of items that should be included in reports of observational studies

	Item No.	Recommendation	Page No.	Relevant text from manuscript
**Title and abstract**	1	(*a*) Indicate the study's design with a commonly used term in the title or the abstract	Title page; Abstract	Title: "...an observational crossover study" Abstract (methods): "We conducted an observational crossover study..."
		(*b*) Provide in the abstract an informative and balanced summary of what was done and what was found	Abstract	Complete structured abstract provided.
**Introduction**				
Background/rationale	2	Explain the scientific background and rationale for the investigation being reported	Introduction	"Virtual patients (VP) are digital educational modalities that enable learners within health professions to interact with patient cases for learning purposes...While VPs offer standardised training opportunities, medical students report that conventional computer-based implementations often lack the authenticity needed for realistic CR training, potentially limiting their educational impact… Given the critical importance of CR skills in clinical practice and the challenges of providing authenticity using traditional VPs, quantitative evaluation is

				essential to determine the effectiveness of emerging VP technologies".
Objectives	3	State specific objectives, including any prespecified hypotheses	Introduction	"This study aimed to compare VP design characteristics that facilitate medical students' training of CR skills, between an AI-enhanced social robotic and a conventional computer-based VP platform, as reported by the students."
**Methods**				
Study design	4	Present key elements of study design early in the paper	Methods	"We conducted an observational crossover study to compare VP design elements that support CR skill training in medical students between an LLM-empowered social robotic platform, i.e., an in-house developed Social AI-enhanced Robotic Interface (SARI) and a computer-based VP platform, i.e., the Virtual Interactive Case system (VIC)…"
Setting	5	Describe the setting, locations, and relevant dates, including periods of recruitment, exposure, follow-up, and data collection	Methods (study design and setting)	"The study was conducted at Karolinska Institutet (KI), a major academic medical university in Stockholm, Sweden. Data collection occurred during mandatory clinical rotations at the Division

				of Rheumatology, Karolinska University Hospital, between the spring term of 2024 and the spring term of 2025.”
Participants	6	(*a*) *Cohort study*—Give the eligibility criteria, and the sources and methods of selection of participants. Describe methods of follow-up<br>*Case-control study*—Give the eligibility criteria, and the sources and methods of case ascertainment and control selection. Give the rationale for the choice of cases and controls<br>*Cross-sectional study*—Give the eligibility criteria, and the sources and methods of selection of participants	Methods (study participants)	“We used convenience sampling, recruiting all sixth-semester medical students who completed rheumatology clinical rotations during the study period. All students (N=421) were invited to participate by completing a survey following their educational experience with both VP platforms. A total of 178 agreed to participate, yielding a response rate of 42.3%. There were no specific inclusion or exclusion criteria. Participation was voluntary”
		(*b*) *Cohort study*—For matched studies, give matching criteria and number of exposed and unexposed<br>*Case-control study*—For matched studies, give matching criteria and the number of controls per case	N/A – crossover design	Not applicable. This is a crossover study where each participant experienced both conditions, serving as their own control, thereby eliminating the need for matching.
Variables	7	Clearly define all outcomes, exposures, predictors, potential confounders, and effect modifiers. Give diagnostic criteria, if applicable	Methods (data collection)	Exposures: Two VP platforms: (1) SARI, and (2) VIC.<br><br>Outcomes: “VP design was evaluated using a validated

				questionnaire with five domains (authenticity, professional approach, coaching quality, learning effects, and overall judgment). Student preferences for CR training were assessed using categorical responses and a visual analogue scale (VAS; 0–10)".<br><br>Confounders: Age, sex, prior VP experience, platform order.
Data sources/ measurement	8*	For each variable of interest, give sources of data and details of methods of assessment (measurement). Describe comparability of assessment methods if there is more than one group	Methods (data collection and statistical analysis)	"Immediately after completion of the virtual outpatient clinic, students who had agreed to participate in this study completed a questionnaire to evaluate the VP platform design, with particular emphasis put on CR training".
Bias	9	Describe any efforts to address potential sources of bias	Methods (opening and statistical analysis)	"Each student served as their own control to minimise between-participant variability"<br><br>Order effects were examined through subgroup analysis /by first platform exposure) and discussed as a limitation.
Study size	10	Explain how the study size was arrived at	Methods (study participants)	Convenience sample; all eligible students during study period

invited (N=421), 178 participated. No formal sample size calculation was conducted. As all eligible students were invited, the study aimed for total inclusion rather than predetermined power.

Continued on next page

Quantitative variables	11	Explain how quantitative variables were handled in the analyses. If applicable, describe which groupings were chosen and why	Methods (statistical analysis)	"For each theme, individual item scores were averaged to create a composite theme score for each student. Theme scores were next compared between platforms. VAS responses from the two platforms were compared to a hypothetical score of 5 denoting equal preference."
Statistical methods	12	(*a*) Describe all statistical methods, including those used to control for confounding	Methods (statistical analysis)	"The Wilcoxon signed-rank test was used to compare responses to Likert-scale data from quantitative themes in the questionnaire described by Huwendiek et al. and VAS responses... The Fisher's exact test with Monte Carlo simulation (10,000 iterations) was used to compare frequencies of categorical responses for VP platform preference based on students' CR training experience."
		(*b*) Describe any methods used to examine subgroups and interactions	Methods (statistical analysis)	Subgroup analyses conducted by sex, prior VP experience, and platform order using the same statistical tests
		(*c*) Explain how missing data were addressed	Methods (statistical analysis)	"Missing data were minimal across all variables. Demographic variables had no missing values (0%) except platform order (1 missing, 0.56%). For Likert scale items evaluating VP design,

				missing data were 1.68% overall (range:0–3.91% per item; maximum 7 missing responses out of 178). VAS preference scores had 1.12% missing (2 of 178 responses), and categorical platform preference had 1.68% missing (3 of 178 responses). Little's MCAR test on Likert data indicated data may not be completely at random (χ^2=157.12; df=108; p=0.001); however, given the very low proportion of missing data (<2% overall) and the paired nature of our crossover design, complete case analysis with pairwise deletion was used."
		(*d*) *Cohort study*—If applicable, explain how loss to follow-up was addressed<br>*Case-control study*—If applicable, explain how matching of cases and controls was addressed<br>*Cross-sectional study*—If applicable, describe analytical methods taking account of sampling strategy	Methods (statistical analysis)	Paired statistical tests (Wilcoxon signed-rank) used to account for crossover design
		(*e*) Describe any sensitivity analyses	Methods (statistical analysis); Discussion	Subgroup analyses and missing data pattern analysis conducted.
**Results**				
Participants	13*	(a) Report numbers of individuals at each stage of study—eg numbers potentially eligible, examined for eligibility, confirmed eligible, included in the study, completing follow-up, and analysed	Methods (study participnts); Results (demographics); Tables 1–2	421 students eligible for recruitment. 178 participated (42.3%). 152–175 with complete data per analysis (n reported in Tables 1–2)

		(b) Give reasons for non-participation at each stage	Methods (study participants)	Participation voluntary. Specific reasons for non-participation was not required or collected.
		(c) Consider use of a flow diagram	Not included	N/A
Descriptive data	14*	(a) Give characteristics of study participants (eg demographic, clinical, social) and information on exposures and potential confounders	Results (demographic and study-specific characteristics)	"Of the 178 students who participated in the questionnaire, 93 (52%) were women and 86 (48%) were men. Most students had no previous experience with VP platforms (150 students, 84%), while 29 (16%) reported prior experience. The mean age was 25.3 (standard deviation: 5.4) years. Regarding platform order, 101 (56%) students started with SARI and 77 (43%) started with VIC."
		(b) Indicate number of participants with missing data for each variable of interest	Methods (statistical analysis); Tables 1–2	"Missing data were minimal across all variables. Demographic variables had no missing values (0%) except platform order (1 missing, 0.56%). For Likert scale items, missing data were 1.68% overall (range: 0–3.91% per item; maximum 7 missing responses out of 178). The varying n values in Tables 1-2 (ranging from 152-175) reflect both missing data AND 'Not applicable' responses being excluded from statistical analyses."
		(c) *Cohort study*—Summarise follow-up time (eg, average and total amount)	N/A	N/A
Outcome data	15*	*Cohort study*—Report numbers of outcome events or summary measures over time	N/A	N/A

		Case-control study—Report numbers in each exposure category, or summary measures of exposure	N/A	N/A
		Cross-sectional study—Report numbers of outcome events or summary measures	Results (all subsections); Tables 1–2; Figures 1–4	"Students rated SARI higher for authenticity of patient encounters (median [IQR]: 4.0 [3.5–4.5] versus 3.0 [2.5–3.5]; p<0.001), professional approach during consultation (median [IQR]: 4.5 [4.0–4.8] versus 4.0 [3.5–4.5]; p<0.001), coaching during consultation (median [IQR]: 4.3 [4.0–4.7] versus 4.0 [3.7–4.7]; p<0.001), learning effect of consultation (median [IQR]: 4.4 [4.0–5.0] versus 4.0 [3.5–4.5]; p<0.001), and overall judgment (median [IQR]: 5.0 [4.0–5.0] versus 4.0 [4.0–5.0]…"
Main results	16	(*a*) Give unadjusted estimates and, if applicable, confounder-adjusted estimates and their precision (eg, 95% confidence interval). Make clear which confounders were adjusted for and why they were included	Results (all subsections); Tables 1–2	All estimates unadjusted (crossover design inherently controls for between-subject confounding). Medians with IQR, test statistics, effect sizes, ORs with 95% CIs provided throughout Results section and Tables 1-2
		(*b*) Report category boundaries when continuous variables were categorized	Methods (data collection)	VP design questionnaire "scored from 1 (strongly disagree) to 5 (strongly agree)". VAS scores "where a lower score denoted a stronger preference for SARI, a higher score denoted a stronger

			preference for VIC, and a score of 5 denoted equal preference”.
	(*c*) If relevant, consider translating estimates of relative risk into absolute risk for a meaningful time period	N/A	N/A

Continued on next page

Other analyses	17	Report other analyses done—eg analyses of subgroups and interactions, and sensitivity analyses	Results (VP-platform preference); Figures 3–4; Supplementary Tables	Subgroup analysis for sex, prior VP experience, and which VP platform students were first introduced to.
**Discussion**				
Key results	18	Summarise key results with reference to study objectives	Discussion (principal findings)	"This observational crossover study aimed to quantitatively compare medical students' perceptions of VP design characteristics that support CR training between an AI-enhanced social robotic platform (SARI) and a conventional computer-based system (VIC). Consistent with our objectives, the results showed that SARI demonstrated consistent superiority across all five domains of VP design, indicating that AI-enhanced social robotic VPs offer significant advantages over conventional computer-based platforms for CR training in medical students."
Limitations	19	Discuss limitations of the study, taking into account sources of potential bias or imprecision. Discuss both direction and magnitude of any potential bias	Discussion (limitations and strengths)	"Firstly, our findings are based on students' perceptions of VP design elements rather than objective measures of CR skill acquisition... Secondly, the single-centre and discipline-specific design within rheumatology may limit the

				generalisability of the findings to other educational contexts or medical specialities. Thirdly, the use of English in case interaction rather than the students' native language (Swedish) may have affected perceived authenticity and interaction quality"
Interpretation	20	Give a cautious overall interpretation of results considering objectives, limitations, multiplicity of analyses, results from similar studies, and other relevant evidence	Discussion (multiple sections)	Findings interpreted in context of educational theory, prior qualitative research, and limitations. Multiple sections discuss cognitive engagement, skill transfer, and comparison with existing literature. Although multiple comparisons were conducted, all analyses were hypothesis-driven and aligned with prespecified domains.
Generalisability	21	Discuss the generalisability (external validity) of the study results	Discussion (limitations and strengths, and implications)	"The single-centre and discipline-specific design within rheumatology may limit the generalisability of the findings to other educational contexts or medical specialities… The study included a substantial sample (n=178) representing 42% of eligible students, which supports the generalisability of the findings within the target population."
**Other information**				
Funding	22	Give the source of funding and the role of the funders for the present study and, if applicable, for the	Funding	"This work was supported by

	original study on which the present article is based		grants from Region Stockholm ALF Pedagogy (FoUI-977096), Karolinska Institutet Pedagogical Project Funding (FoUI-964139), the Swedish Rheumatism Association (R-1013624), King Gustaf V's 80-year Foundation (FAI-2023-1055), Swedish Society of Medicine (SLS-974449), Nyckelfonden (OLL-1023269), Professor Nanna Svartz Foundation (2021-00436), Ulla and Roland Gustafsson Foundation (2024-43), Region Stockholm (FoUI-1004114), and Karolinska Institutet."

*Give information separately for cases and controls in case-control studies and, if applicable, for exposed and unexposed groups in cohort and cross-sectional studies.

Note: An Explanation and Elaboration article discusses each checklist item and gives methodological background and published examples of transparent reporting. The STROBE checklist is best used in conjunction with this article (freely available on the Web sites of PLoS Medicine at http://www.plosmedicine.org/, Annals of Internal Medicine at http://www.annals.org/, and Epidemiology at http://www.epidem.com/). Information on the STROBE Initiative is available at www.strobe-statement.org.
