# Supplementary material for: AI-Enhanced Social Robotic Versus Computer-Based Virtual Patients for Clinical Reasoning Training in Medical Education: Observational Crossover Cohort Study
Source: J Med Internet Res. 2025 Nov 27;27:e82541. doi: 10.2196/82541 (PMC12699248; doi:10.2196/82541)
Supplement: Multimedia Appendix 4 [file jmir_v27i1e82541_app4.pdf]

**BRIEF NAME**	
1. INTERVENTION: Provide a brief description of the educational intervention for all groups involved [e.g. control and comparator(s)].	All sixth-semester medical students at Karolinska Institutet experienced two virtual patient (VP) platforms during mandatory rheumatology clinical rotations: (1) the Social AI-enhanced Robotic Interface (SARI), which combines a Furhat social robot with a large language model from OpenAI (GPT-3.5 turbo), and (2) the Virtual Interactive Case system (VIC), a conventional computer-based VP platform. Students interacted with 9 unique rheumatology cases in total (5 cases on one platform, 4 on the other) over 1,5 days, working in pairs or small groups of three, followed by case-specific seminars led by consultant rheumatologists.
**WHY - this educational process**	
2. THEORY: Describe the educational theory (ies), concept or approach used in the intervention.	The educational intervention was grounded in experiential learning theory [1], where students learn through direct engagement with simulated clinical scenarios. The intervention also incorporated principles of situated learning and authentic learning environments, emphasising the importance of realistic clinical contexts for developing clinical reasoning (CR) skills. The design recognised that effective CR development requires active cognitive engagement with patient cases that mirror real clinical encounters. The collaborative learning approach (pairs or small groups of three students) was based on evidence that interaction and active collaboration favour CR training [2].
3. LEARNING OBJECTIVES: Describe the learning objectives for all groups involved in the educational intervention.	The learning objectives for all students participating in the virtual outpatient clinic were: (1) To gather a structured and relevant medical history from a patient with a rheumatic condition, (2) to formulate and think about relevant differential diagnoses for rheumatological presentations, (3) to practise CR processes in controlled, safe environments that simulate real clinical encounters, and (4) to develop diagnostic decision-making skills appropriate for sixth-semester medical students.
4. EBP CONTENT: List the foundation steps of EBP (ask, acquire, appraise, apply, assess) included in the educational intervention.	Not applicable. This intervention focused on CR skill development through VP interactions rather than teaching evidence-based practice methodology.

**WHAT**	
5. MATERIALS: Describe the specific educational materials used in the educational intervention. Include materials provided to the learners and those used in the training of educational intervention providers	Students received written information on generic medical history questions typically used at the rheumatology outpatient clinic, organised by organ system with example questions. They also received case-specific contextual information for each case, including laboratory test results with reference values. This supplementary information was provided in written form before SARI cases and incorporated into VIC cases.<br><br>Seminar leaders received a framework for follow-up seminars with example topics to discuss for each VP.
6. EDUCATIONAL STRATEGIES: Describe the teaching/learning strategies (e.g. tutorials, lectures, online modules) used in the educational intervention.	VP interactions in small groups or dyads allowed for interaction and active collaboration, and peer discussion during CR processes.<br><br>Case-specific follow-up seminars following completion of each VP case to discuss case information, pose questions and summarise cases in a structured manner to practise clinical communication and propose management recommendations.
7. INCENTIVES: Describe any incentives or reimbursements provided to the learners.	Students received no financial compensation or academic credits for study participation beyond the mandatory educational activity. Participation in the virtual outpatient clinic was a mandatory component of their rheumatology clinical rotation, but completion of the research questionnaire was voluntary.
**WHO PROVIDED**	
8. INSTRUCTORS: For each instructor(s) involved in the educational intervention describe their professional discipline, teaching experience/expertise. Include any specific training related to the educational intervention provided for the instructor(s).	Two consultant rheumatologists at the Division of Rheumatology, Karolinska University Hospital, alternated weekly to lead the case-specific follow-up seminars. Both instructors had prior teaching experience within the medical programme and had received tutoring in supervision prior to the clinical course. Instructors received standardised instructions for seminar facilitation, including a structured framework for case discussions while maintaining flexibility for open dialogue. They participated in continuous monitoring discussions before and after seminars to ensure implementation fidelity of the intervention.

**HOW**	
9. DELIVERY: Describe the modes of delivery (e.g. face-to-face, internet or independent study package) of the educational intervention. Include whether the intervention was provided individually or in a group and the ratio of learners to instructors.	Face-to-face delivery in pairs or small groups of 2–3 students for VP interactions, followed by group seminars of 4–9 students (2–3 student groups combined) after completing each case.
**WHERE**	
10. ENVIRONMENT: Describe the relevant physical learning spaces (e.g. conference, university lecture theatre, hospital ward, community) where the teaching/learning occurred.	The virtual outpatient clinic at the Division of Rheumatology, Karolinska University Hospital, Stockholm, Sweden. VP interactions were undertaken at seminar rooms at the hospital clinic.
**WHEN and HOW MUCH**	
11. SCHEDULE: Describe the scheduling of the educational intervention including the number of sessions, their frequency, timing and duration.	One and a half days total. Students experienced 9 unique VP cases (one case was identical across both platforms). Platform order was determined by clinical rotation scheduling.
12. Describe the amount of time learners spent in face to face contact with instructors and any designated time spent in self-directed learning activities.	30 minutes allocated per VP case and 15 minutes of follow-up seminars.
**PLANNED CHANGES**	
13. Did the educational intervention require specific adaptation for the learners? If yes, please describe the adaptations made for the learner(s) or group(s).	No specific adaptations were required for individual students or groups. All students received the same support. The primary planned implementation was to develop all VP cases in English to ensure accessibility for international exchange students. Cases were designed with difficulty levels adapted to the sixth-semester medical curriculum at Karolinska Institutet, representing a planned adaptation to the learner level rather than to specific learner groups.
**UNPLANNED CHANGES**	
14. Was the educational intervention modified during the course of the study? If yes, describe the changes (what, why, when, and how).	No modifications were made to the intervention content during the study period. Some minor technical improvements to the SARI platform may have occurred during implementation that did not affect case content, clinical scenarios, or educational delivery. The core

	educational components remained consistent throughout the study period.
**HOW WELL**	
15. ATTENDANCE: Describe the learner attendance, including how this was assessed and by whom. Describe any strategies that were used to facilitate attendance.	421 sixth-semester medical students participated in the educational activities and were invited to participate in the study during their mandatory rheumatology clinical rotations between Spring 2024 and Spring 2025. 178 students (42.3 %) agreed to participate by completing the questionnaire and signing an ICF prior to enrolment.
16. Describe any processes used to determine whether the materials (item 5) and the educational strategies (item 6) used in the educational intervention were delivered as originally planned.	Consultant rheumatologists had received standardised instructions for seminar facilitation, and there was continuous monitoring of implementation fidelity through discussions before and after seminars. Feedback from these discussions resulted in notes and slight technical and logistical improvements that did not affect the intervention contents but allowed for implementation fidelity.<br><br>The implementation was monitored through regular discussions between the research team and seminar leaders, ensuring that educational materials and strategies were delivered consistently throughout the study period.
17. Describe the extent to which the number of sessions, their frequency, timing and duration for the educational intervention were delivered as scheduled (item 11).	No major deviations from the planned schedule occurred. All students completed all nine VP cases as intended over the 1.5-day period and participated in all of the 9 follow-up seminars. The 30 minute-allocation per VP case interaction and 15-minute follow-up seminars were maintained consistently across all student groups. Minor variations in exact timing may have occurred due to the self-directed nature of VP case exploration, technical issues, and logistic reasons, but the overall structure and sequence remained as planned throughout the study period from Spring 2024 to Spring 2025.

References

1. Yardley, S.; Teunissen, P.W.; Dornan, T. Experiential learning: transforming theory into practice. *Med Teach* **2012**, *34*, 161-164, doi:10.3109/0142159x.2012.643264.
2. Edelbring, S.; Parodis, I.; Lundberg, I.E. Increasing reasoning awareness: video analysis of students' two-party virtual patient interactions. *Jmir Med Educ* **2018**, *4*, e9137.
